# Development of a low-coverage whole genome sequencing screen for apomixis using a diverse set of *Malus* germplasm

**DOI:** 10.1371/journal.pgen.1012289

**Published:** 2026-09-08

**Authors:** Charity Z. Goeckeritz, Václav Polcar, Benjamin Gutierrez, Tomáš Urfus, Alex Harkess

**Affiliations:** 1 HudsonAlpha Institute for Biotechnology, Huntsville, Alabama, United States of America; 2 Department of Botany, Faculty of Science, Charles University, Praguea, Czech Republic; 3 United States Department of Agriculture, Geneva, New York, United States of America; The University of Kansas, UNITED STATES OF AMERICA

## Abstract

In the past decade, plant biologists have made several major discoveries pertaining to the genetic basis of apomixis (clonal propagation by seed) that have shown promise in preserving high-value hybrid rice and sorghum genotypes. This progress was made possible by foundational gene discovery efforts in model species and natural apomicts, but pleiotropic obstacles still limit its broad agricultural adoption, especially in eudicots. Thus, it follows that investigations of novel apomicts should lead to the development of new molecular tools for plant breeding. The two most common ways to identify clonal seed production are flow-cytometry seed screens and genome sequencing to compare the DNA sequences of the maternal parent and progeny, traditionally using low-throughput markers. While flow-cytometry has been the dominant method for more than two decades, it provides indirect information on the genetics of a resulting embryo and can be ineffective in certain species. Here we developed a method using short-read whole-genome sequencing at moderately low coverage (averaging 3X and 6X) to screen diverse *Malus* genotypes maintained in a USDA germplasm collection for clonal seed production. In total, we sequenced 55 genotypes, 1,216 of their embryos, and identified 17 previously undescribed apomictic genotypes. Several more were detected with the flow cytometry seed screen, which helped resolve certain types of reproduction and sources of noise in low-coverage datasets. This low-pass screening-by-sequencing method is a relatively low-cost, rapid method for detecting apomictic genotypes in diverse plant germplasm and when used thoughtfully in conjunction with flow cytometry, provides a new way to visualize the genetic outcomes of sexual and asexual reproduction in plants.

## Author summary

The most established method to determine whether a plant produces clonal seed is the flow cytometry seed screen. However, this method cannot be applied to some species and circumstances and does not directly test for clonality. Therefore, we explored the utility of a high throughput, low-pass whole genome sequencing screen at variable levels of coverage to directly test whether *Malus* embryos are clones of their maternal genotype. We present a simple way to visualize the outcomes of plant reproduction with low-pass sequencing data, consider the ways in which flow cytometry and sequencing complement one another, and discuss how advancing technologies will enable sequencing to become a more routine method to study reproductive plant biology.

## Introduction

Apomixis, or clonal propagation via seed, is a complex reproductive process that has evolved many times in Angiosperms [1]. In gametophytic apomicts, apomixis is characterized by the absence of gamete reduction, recombination, and segregation (apomeiosis), and the spontaneous development of an embryo without fertilization (parthenogenesis) [2]. Apomixis has been documented in several hundred genera, including those of direct economic value (e.g., *Rubus*, *Malus*, *Cucumis*) and crop wild relatives (e.g., *Tripsacum* for maize) [3–6]. Identifying the genes underlying apomixis has been a highly sought-after goal for plant biologists for decades, as these genes have the potential to revolutionize plant breeding. Not only do the genes controlling natural and synthetic apomixis allow for the preservation of valuable genotypes, but they are also being investigated for their capacity to create haploids and to strategically engineer heterotic polyploids [7–10].

The field has had several major breakthroughs in the past decade with the characterization of *BABYBOOM* (*BBM*) in *Cenchrus squamulatus* (previously *Pennisetum squamulatum*) and *PARTHENOGENESIS* (*PAR*) in *Taraxacum officinale*, both of which are sufficient to induce parthenogenesis in gametophytic apomicts [11,12]. To date, no genes from natural apomicts have been formally characterized for apomeiosis, although simultaneous knockouts of several genes regulating meiosis has been shown to create a synthetic version termed *MiMe* (*Mitosis instead of Meiosis*) [13,14]. Using advanced CRISPR technologies to combine the parthenogenesis genes discovered in natural apomicts with the *MiMe* system, synthetic apomixis has become highly efficient in rice and its agricultural application is now on the horizon for this crop [15–20]. More recently, researchers also successfully engineered highly penetrant synthetic apomixis in two sorghum hybrids [21]. Still, the applicability of *MiMe* to other systems remains unclear, especially in eudicot species. Several works have already demonstrated alternative genes to *SPO11*, *REC8,* and *OSD1* are necessary to abolish meiosis in other plants while minimizing pleiotropic effects, likely due to gene expansion, contraction, and the functional divergence of these homologs throughout evolution [9,22,23]. However, the prevalence of apomixis in flowering plants means ample opportunity to discover new tools for plant breeding [1,24].

The discovery of these genes has been slowed by a number of factors. Traditionally, gene discovery requires the generation of segregating populations, yet apomixis is partly defined by the absence of meiosis and recombination. While most natural apomicts are facultative and reproduce sexually at a rate dependent on genotype and environment [25–28], fine mapping these genes has still been hindered by long generational times, polyploidy, distorted segregation, and large hemizygous, non-recombining regions [29,30]. Fortunately, advancing genomic technologies are beginning to offer alternative approaches to complement conventional ones. Wang *et al*. (2022) used resequencing data of diverse genotypes to fine map *RWP-RK* genes in *Citrus* and *Fortunella*, and Yadav *et al*. (2023) identified an orthologous gene in *Mangifera*. This gene had previously been confirmed in *Citrus* to induce somatic embryogenesis [31,32]. Notably, both examples mapped genes in diploids exhibiting sporophytic apomixis, which tends to segregate normally as a single dominant trait. Genomic methods will be especially impactful in perennial species with loci embedded in larger, non-recombining regions of the genome (e.g., aposporic gametophytic apomicts) by taking advantage of ancestral recombination to narrow in on gene candidates. These studies also underscore the enormous value of maintaining diverse, living collections of adult plants and developing international collaborations to advance our understanding of the evolution and functional genomics of plant reproductive biology.

A number of methods have been developed to screen for apomixis, including microscopy techniques to observe megagametogenesis and embryology, pollen exclusion and pistil decapitation for autonomous apomixis, the flow cytometric seed screen (FCSS), and the comparison of a variety of genetic markers [33]. The most popular method to distinguish apomixis, sexuality, and intermediate forms of reproduction is the FCSS, as it is moderate-throughput and ploidy agnostic [34,35]. The method indirectly infers reproductive information by examining the ploidy of the embryo and endosperm within a seed, which differ in flowering plants due to the process of double fertilization. The ploidy ratio for these two tissues is approximately 2: 3 for sexually-reproducing individuals, but deviates substantially for individuals capable of apomixis [34–36]. For example, in a tetraploid gametophytic apomict with a typical polygonum-type embryo sac, all cells of the embryo sac including the egg and central cells are expected to be unreduced (4C). The clonal egg cell may undergo parthenogenesis, allowing both sperm cells of a pollen tube to fuse with the central cells and participate in endosperm formation. If a pollen parent of the same ploidy produces reduced gametes, the ploidy of the endosperm would be 4C^*m*^ + 4C^*m*^ + 2C^*p*^ + 2C^*p*^, with *m* and *p* indicating the maternal and paternal genome contributions, respectively. The resulting ratio between embryo and endosperm in this hypothetical scenario would be 4: 12. As long as sufficient nuclei from these two tissues are intact, FCSS would conclude this seed was produced through pseudogamous apomixis. However, the embryo is not directly confirmed to be clonal, resulting in some uncertainty when interpreting the results (e.g., it would not capture recombination if it had occurred).

Indeed, there are circumstances in which plants may produce unreduced gametes that are not truly clonal through meiotic restitutional processes [37]. For instance, individuals capable of first division restitution (FDR) may produce unreduced male or female gametes that are not identical to the somatic chromosomes of the parent if homologous crossovers occurred during meiosis I [38,39]. Conversely, plants exhibiting second division restitution (SDR), where sister chromatids fail to separate during meiosis II, may produce mostly homozygous unreduced gametes that differ from the seed parent [40,41]. Although these events vary in frequency according to genotype and environmental factors, they should be considered possibilities before assuming embryos are maternal clones based on FCSS alone. FCSS can also be uninformative or technically challenging in specific species, such as pseudogamous apomicts with uninucleate maternal endosperm contributions or species with diminished endosperm [34,42,43]. For example, Ptáček *et al*. recently conducted FCSS on several thousand seeds from North and South American alpine species, but 67.6% of the seeds lacked measurable endosperm or were aborted and the reproductive mode could not be determined [43]. FCSS also requires specialized equipment and expertise, which can become prohibitively expensive when outsourced as a service. Therefore, it is prudent to develop additional high-throughput and low-cost methods for screening apomixis to complement current ones.

Traditional sequencing-based approaches for screening progeny or inferring apomixis in populations typically utilize a handful of polymorphic markers [33,44]. The likelihood that individuals in geographically disjunct populations share identical sets of multi-locus genotypes is low even for a dozen unlinked markers, thus apomixis can be inferred [45,46]. However, using very few markers may lead to erroneous conclusions depending on population structure, so additional unlinked genetic markers or other complementary data (such as FCSS) may be necessary to resolve reproductive types [47,48]. While screening for apomixis through sequencing is the most direct method, it is not yet routine due to the costs and labor associated with these methods, including planting seed, DNA extraction, library preparation, and the sequencing itself. We expect this to change as DNA extraction has the potential to be automated, library preparation kits now allow for the multiplexing of hundreds of samples, and sequencing costs continue to decline. For example, low-pass whole-genome sequencing (WGS) may be a promising approach to detect clonality, especially when FCSS fails to distinguish reproductive types or when a more direct means of detecting clonality is desired. While samples are sequenced to shallow overall coverage across the genome (between 0.01X - 5X), random sites in the genome may be captured at higher depths than average and can thus be called confidently [49,50]. For breeding applications, the dispersed sites across the genome are considered for the population, and imputation is used to fill missing regions prior to associating traits with variation [51,52].

For the present study, we were interested in whether low-pass sequencing would produce enough high-confidence data to detect clonality, selfing, outcrossing, and other forms of reproduction in a highly heterozygous genus. We selected apple (*Malus*, Rosaceae) for testing a low-pass WGS screen given the availability of adult germplasm maintained by the USDA, its amenability to FCSS as a complementary approach, and the well-supported accounts of diverse types of reproduction. For instance, wild *Malus* species native to eastern Asia and North America have previously been documented to exhibit aposporic apomixis according to cytological and flow cytometry analyses [4,33,53–56]. Rosaceae apomicts are considered pseudogamous apomicts, with pollination being required to fertilize the central nuclei for endosperm formation [25,54]. However, certain studies have used pistil decapitation to test for apomictic capacity, suggesting autonomous endosperm formation may also occur in *Malus* [25]. Both sequencing and FCSS approaches have confirmed progeny may be derived from the fertilization of unreduced gametes or haploid parthenogenesis, which result in increased or decreased ploidy in the next generation, respectively [44,57]. Specifically, foreign pollination of unreduced clonal egg cells are referred to as B_III_ hybrids [58]. Finally, triploid accessions of *M. × domestica* have been documented to produce unreduced and aneuploid gametes through aberrant sexual processes [59,60].

We generated whole-genome sequencing data for 55 *Malus* genotypes to at least 15X (herein referred to as the ‘maternal genotypes’), as well as 1,216 of their embryos at targets of 3X or 6X whole genome coverage. Actual coverage per sample varied, allowing us to examine the relationship between the number of high-quality sites captured and sequencing coverage. These genotypes represent individuals from 22 of the 55 species recognized by Phipps *et al*. 1990 and a number of cultivated hybrids, ranging in ploidy from diploid to tetraploid [61,62]. We developed a simple analysis workflow to compare informative biallelic SNPs and detect clonally-produced embryos. FCSS was conducted on a subset of the sequenced genotypes (n = 26) to cross-validate these data types and understand how they complement one another. Our study is the first to establish low-pass whole-genome sequencing as a scalable screening strategy for apomixis. We discuss the advantages and limitations of the method, including ways to make it more efficient and affordable.

## Results

### Sequencing statistics and adjusting experimental coverage

**Fig 1:**
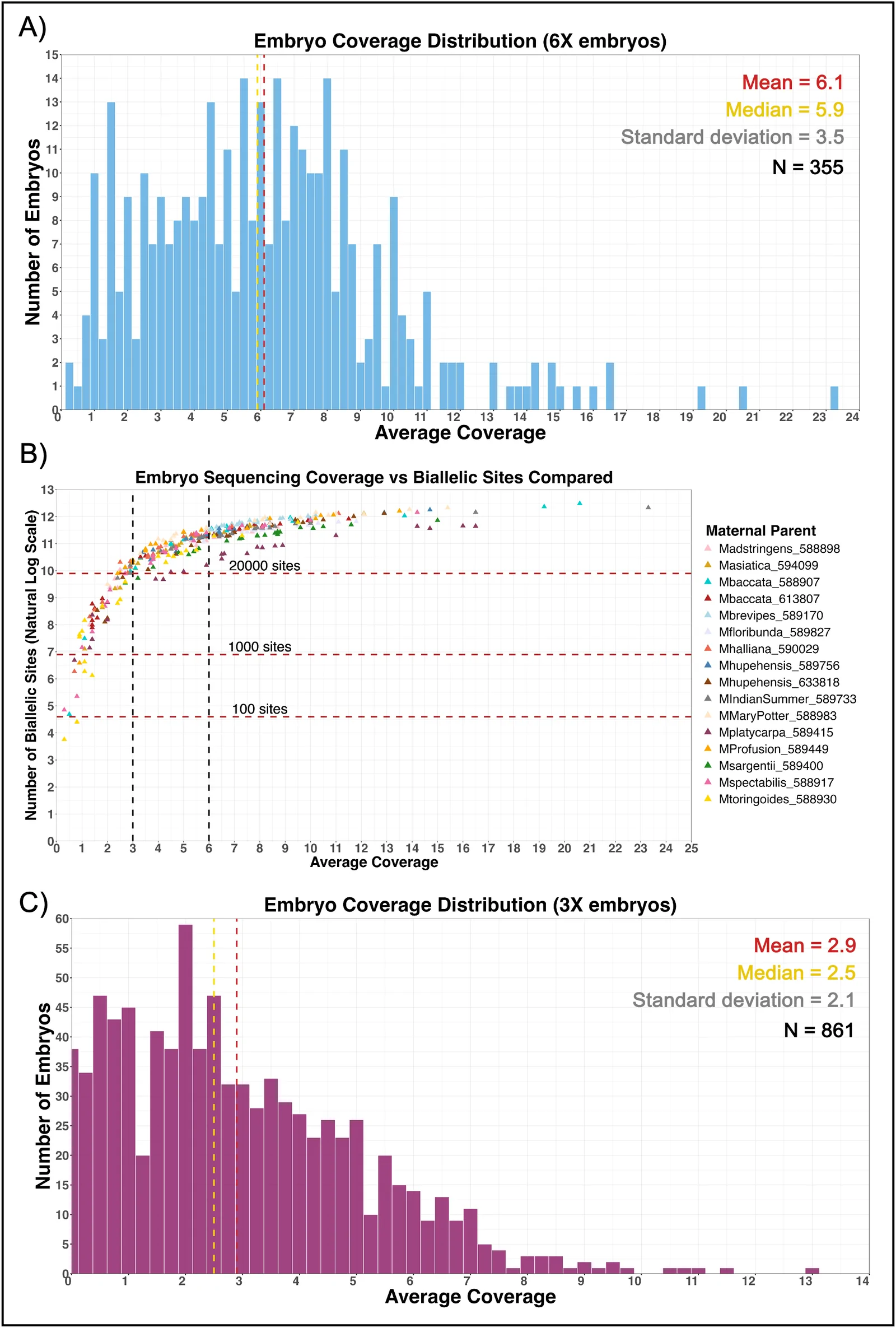
Descriptive statistics for 1,216 total *Malus* embryos sequenced to 6X and 3X average whole genome coverage. A) Distribution of average coverages for all embryos that were sequenced to 6X (up to 96 samples per lane).Average coverage was calculated as (# of fastp-processed reads aligned to the reference genome)*150/ 650000000), or the number of trimmed aligned reads (prior to quality filtering) multiplied by the read length format, then divided by a typical monoploid genome size for *Malus*. Bin size = 0.25. **B)** Relationship between average sequencing coverage and the number of high-quality, biallelic variant sites compared between an embryo and its maternal parent (natural log scale). The target coverages of the present study are marked (3X, 6X) in addition to y-values corresponding to 100, 1000, and 20,000 sites compared. **C)** Distribution of average coverages for all embryos that were sequenced to 3X (up to 192 samples per lane). Average coverage for each embryo was calculated similarly to those sequenced at 6X. Bin size = 0.25.

To develop a low-pass WGS screen for apomixis, we first sequenced *Malus* embryo DNAs from 16 maternal genotypes (n = 355) to an average coverage of 6X (Fig 1A). We examined coverage variation in the 6X embryo sequencing data to determine the relationship between sequencing coverage of the embryo and the number of high-quality biallelic sites that were captured for comparison to the maternal parent (Fig 1B). A biallelic site was considered high-quality if it passed our stringent quality filters, which were determined by optimizing the variant calling error rate with downsampled maternal libraries (See Materials & Methods and S1 Fig). Given the association between captured sites and sequencing coverage, we reasoned embryos sequenced at an average coverage of 3X would still provide hundreds to thousands of informative sites to distinguish clonality from other modes of reproduction. Notably, even lower coverages (e.g., 1.5X) were estimated to provide over 1000 sites for comparison between the maternal genotype and embryo. However, we moved forward with 3X WGS coverage per embryo to minimize sample drop-out (defined as a sample not sequenced deeply enough to provide at least 50 heteroallelic and 50 homoallelic sites for comparison). Embryos from 39 additional maternal genotypes were subsequently sequenced at an average of 3X coverage (n = 861; Fig 1C and S1 Table). To confirm the compared sites were distributed along the lengths of the 17 *Malus* reference chromosomes, locations from one embryo of the lowest coverage and one for the average coverage were plotted for *M. hupehensis* 633818 and *M. spectabilis* 588917 (S2 Fig).

### Descriptive results of biological and technical controls

**Fig 2:**
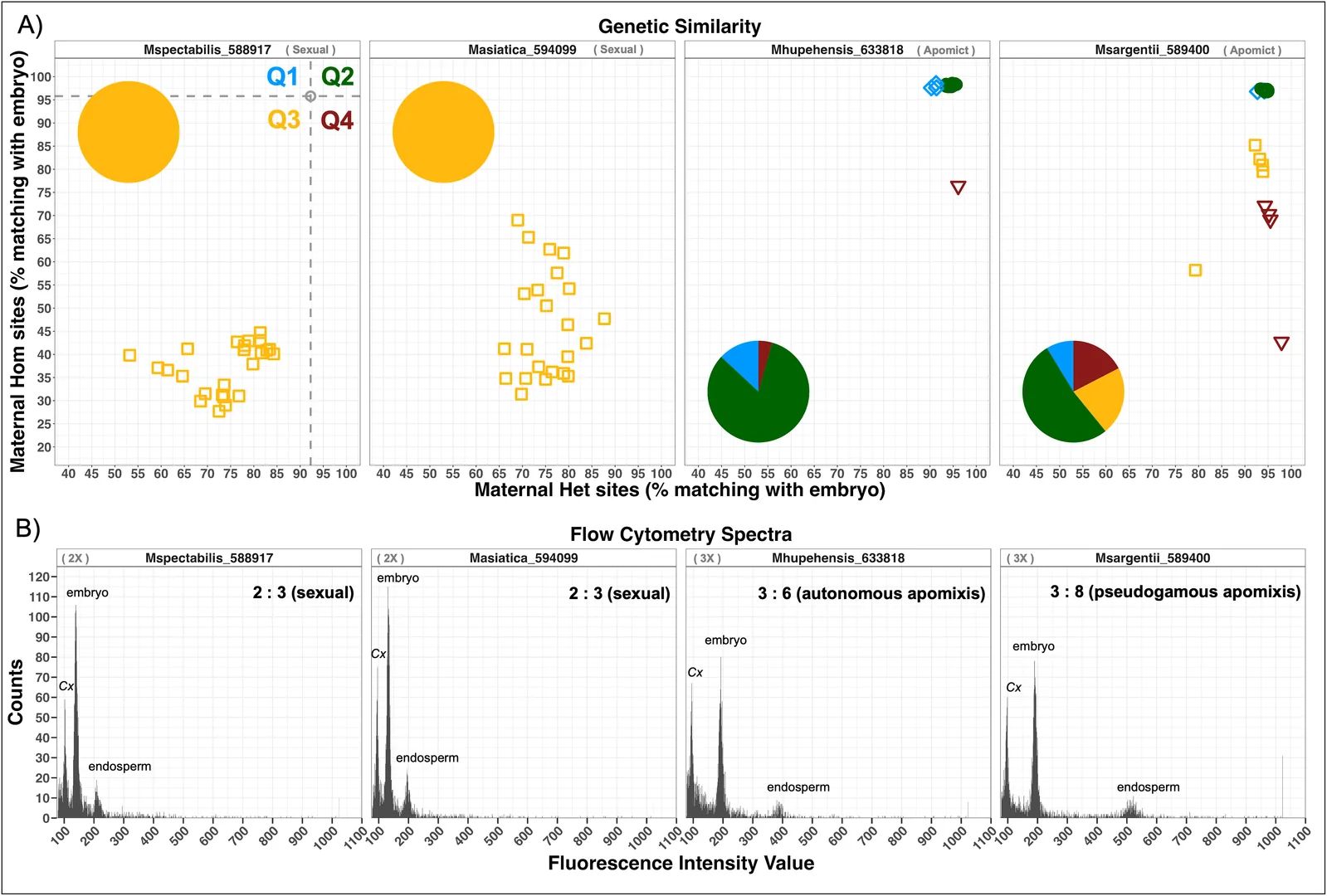
Screening results for genotypes known to reproduce through sexual processes, specifically outcrossing (*M. spectabilis* 588917 and *M. asiatica* 594099) and facultative apomixis (*M. hupehensis* 633818 and *M. sargentii* 589400). **A)** Plots showing the genetic similarities for each embryo and the maternal parent. The x-axis represents % matching sites where the maternal parent was called heterozygous (0/1) while the y-axis represents % matching sites where the maternal parent was called homozygous (0/0 or 1/1). Each open symbol represents one embryo. The clonal boundaries for each embryo were determined based on its sequencing coverage and a quantile regression model with tau = 0.05. Boundaries for a hypothetical embryo at 1X sequencing coverage are shown on the *M. spectabilis* 588917 subplot with dashed gray lines. The quadrants these boundaries delineate are noted, and the reproductive scenarios they represent are described in the main text. Blue diamonds = Q1, green circles = Q2, yellow squares = Q3, red inverted triangles = Q4. A pie graph is included within one corner of each subplot to show the proportion of embryos falling in each quadrant for that respective maternal parent. **B)** A representative flow cytometry spectrum from each genotype showing the predominant mode of reproduction. The internal standard (*Cx*), embryo, and endosperm peaks are labeled in each subplot. The approximate ratio of embryo to endosperm ploidy is given with the predicted mode of reproduction for the seed near the top right. Ploidy is also given in the top left corner of each subplot.

Among the 16 maternal genotypes with embryos sequenced to 6X coverage were controls for reproductive modes, including *M. spectabilis* 588917 and *M. asiatica* 594099 for obligate sexual reproduction through outcrossing (xenogamy), and *M. hupehensis* 633818 and *M. sargentii* 589400 for facultative apomixis (Fig 2A). The genetic differences between a maternal genotype and each of its embryos were visualized as a percent of matching sites, split on each axis by whether a site was originally homozygous (0/0 or 1/1; y-axis in Fig 2A), or originally heterozygous (0/1; x-axis in Fig 2A) in the maternal parent. This way we could quickly visualize the addition of new alleles (outcrossing or fertilization from foreign pollen) on the y-axis and recombination and/ or the segregation of gametes on the x-axis. As expected, sexual controls (*M. spectabilis* 588917 and *M. asiatica* 594099) produced embryos with dispersed similarities compared to the maternal genotype, reflective of sexual outcrossing. Facultative apomixis controls (*M. hupehensis* 633818 and *M. sargentii* 589400) showed most embryos clustering with nearly 100% similarities on both axes, representing apomixis. All seeds analyzed by FCSS in 2023 and 2024 for the sexual controls were indicative of sexual reproduction (embryo: endosperm DNA quantities were approximately 2: 3), with the exception of one seed from *M. asiatica* 594099. This seed (1 of 10 analyzed) was inferred to have been produced by autonomous apomixis, with an embryo: endosperm ratio of 2: 4. FCSS data analyzed for the facultative apomixis controls indicated a mix of seed produced through pure apomixis (an unreduced egg cell + parthenogenesis and either autonomous or pseudogamous endosperm formation), B_III_ hybridization or selfing, and sex (Fig 2B and Tables 1 and S2).

**Table 1:** Flow cytometry seed screen (FCSS) summary for 26 Malus genotypes. Taxon = Genotype and PI number in the USDA germplasm database; Sexuality = seed counts exhibiting embryo:endosperm (Emb:End) ratios reflective of syngamy/ sexual reproduction. Here, this includes gamete reduction + fertilization or genome increase scenarios, where unreduced eggs are fertilized with self or foreign pollen (B_III_ selfing or hybridization). Pseudogamous apomixis = seed counts exhibiting Emb: End ratios reflective of pseudogamous apomixis, where ploidy of the embryo is the same as the maternal parent and endosperm is (2*Emb + Paternal contribution). Autonomous apomixis = seed counts exhibiting Emb: End ratios reflective of autonomous apomixis, where ploidy of the embryo is the same as the mother and the endosperm is (2*Emb). Haploid parthenogenesis = seed counts exhibiting Emb: End ratios reflective of haploid parthenogenesis, where ploidy of the embryo is half the maternal parent’s and the endosperm is (2*Emb + Paternal contribution). Total Seed Analyzed = total seeds analyzed by FCSS in both years (2023 and 2024). See S3 Table for more information. * = potential uninucleate endosperm formation.

Taxon	Sexuality	Apomixis		Haploid parthenogenesis	Total Seed Analyzed
		Pseudogamous apomixis	Autonomous apomixis		
*Malus* × *adstringens* 588898	55	1	1	0	57
*Malus* × *brevipes* 589170	40	3	1	0	44
*Malus asiatica* 594099	29	0	1	0	30
*Malus baccata* 588907	33	0	0	0	33
*Malus baccata* 613807	7	3	12	0	22
*Malus coronaria* 589344	12	1	1	0	14
*Malus coronaria* 589977	14	4	0	0	18
*Malus coronaria* 589988	6	8	0	0	14
*Malus coronaria* 590000	8	6	0	0	14
*Malus coronaria* 590014	20	0	0	0	20
*Malus floribunda* 589827	36	0	0	0	36
*Malus halliana* 590029	30	0	0	0	30
*Malus hupehensis* 589756	18	0	0	0	18
*Malus hupehensis* 633818	0	0	10	0	10
*Malus* ‘Indian Summer’ 589733	44	0	0	0	44
*Malus ioensis* 590008	20	0	0	0	20
*Malus* ‘Marry Potter’ 588983	58	0	0	0	58
*Malus platycarpa* 588752	19	4	1	0	24
*Malus platycarpa* 588847	9	3	0	1	13
*Malus platycarpa* 589198	12	8	0	0	20
*Malus platycarpa* 589356	19	3	0	0	22
*Malus platycarpa* 589415	4	14	0	0	18
*Malus* ‘Profusion’ 589449	36	0	1	0	37
*Malus sargentii* 589400	5	2	0	0	7
*Malus spectabilis* 588917	20	0	0	0	20
*Malus toringoides* 588930	19	2*	3	1	25

One genotype in our initial experiments (*M. platycarpa* 589415) showed stark disagreement between its FCSS and low-pass sequencing results – while the FCSS results suggested this genotype is a facultative apomict, the sequencing data suggested the embryos were produced only through sexual processes. We considered this could be due to 1) environmental differences affecting reproduction between the 2023 and 2024 fruiting years, 2) possible technical error related to comparing different library types (SeqWell/ NEBNext for the maternal parent to Twist for the embryos), or 3) an unknown biological reason specific to this genotype.

To distinguish between these scenarios, *M. platycarpa* 589415 embryo DNAs were also sequenced for the same year as seeds used for FCSS (2024). Further, additional controls were incorporated into all subsequent 3X embryo coverage experiments. Specifically, we prepared libraries for 1 – 3 maternal DNA extracts in parallel with its embryos. These maternal ‘spike-ins’ allowed us to partition technical error rates specific to certain genotypes, including from the comparisons of different library types. They also theoretically emulated clonal embryos in the absence of biological noise (e.g., somatic mutations). Examination of % matching sites between the spike-ins and the deeply-sequenced maternal library revealed a clear bimodal distribution for both heteroallelic and homoallelic sites, with spike-ins derived from *M. platycarpa* 589415 and three other tetraploid *M. platycarpa* accessions showing substantially lower % matches (S3 Fig). To more thoroughly understand the reason(s) for this, the pair of spike-ins from each of the four tetraploid *M. platycarpa* genotypes and three other pairs of spike-ins from three other tetraploids (*M. coronaria* 589344, *M. sikkimensis* 589599, and *M. sargentii* 589372) were compared within genotype and Twist library preparation as they were all sequenced to adequate coverage (> 3.7X). Despite being prepared from the same DNA extract and same library kit, *M. platycarpa* spike-in comparison similarities still lagged 7–8% lower than other genotypes, even when controlling for ploidy. We therefore conclude the larger discrepancies seen only in the tetraploid *M. platycarpa* spike-ins are due to an unknown biological reason inherent to the genotypes themselves.

### Establishing clonal thresholds with quantile regression

To statistically distinguish clonal embryos from other modes of reproduction, quantile regression was used to estimate the 5^th^ percentile (tau = 0.05) of heteroallelic and homoallelic percentage cutoffs. The boundaries for tetraploid *M. platycarpa* embryos were modeled separately from all other genotypes. Biological controls (true apomictic embryos from *M. hupehensis* 633818 and *M. sargentii* 589400; n = 33) and technical controls (maternal spike-ins; n = 77) were pooled only after verifying these two groups’ error rates responded similarly to changes in coverage. Coverage and experiment type (biological or spike-in) significantly influenced the 5^th^ percentile threshold for heteroallelic sites (p < 0.05). For approximately 1X increase in raw sequencing coverage, the 5^th^ percentile threshold increased by 0.26%. Spike-ins performed slightly better than biological controls by 1.04%; this was expected as biological controls encompassed additional sources of noise such as somatic mutations. Coverage and experiment type did not significantly affect the 5^th^ percentile threshold for homoallelic sites (p > 0.05). Therefore, contrary to the heteroallelic boundary that shifted according to sequencing coverage, a single homoallelic match threshold (= 95.8%) was applied to most embryos.

The four tetraploid *M. platycarpa* genotypes clearly showed a complex error distribution distinct from all other genotypes, emphasizing the need to establish unique clonal thresholds (S3 Fig). The heteroallelic and homoallelic boundaries for the tetraploid *M. platycarpa* embryos were established using quantile regression and their spike-in controls, with a coverage-based model for the heteroallelic threshold and intercept-only model for the homoallelic threshold (= 82.1%). The spike-ins from the tetraploid *M. platycarpa* genotypes showed higher error rates than biological controls, making it difficult to model biological noise in these samples. To compensate for this, an additional biological penalty was applied to these samples’ heteroallelic boundary based on the model built for all other genotypes.

### Descriptions of the low-pass sequencing quadrants

Establishing the boundaries for clonal embryos effectively separated sequenced embryos into four quadrants according to their type of reproduction (Fig 2A; *M. spectabilis* 588917 subplot). The FCSS data further strengthened our conclusions for deducing the reproductive biology scenarios represented by the sequencing quadrants. Q1 represented gamete reduction, segregation, recombination, and self-pollination or autogamy. There was also the possibility that embryos produced by haploid parthenogenesis could appear in Q1 (e.g., AABB → BB and AAAA → AA). However, only two haploid parthenogenesis events, both in tetraploids, were captured with FCSS and thus this type of reproduction may be rare in *Malus* (Tables 1 and S3). Q2 represented apomixis, or true clonal seed produced by both apomeiosis and parthenogenesis. Importantly, we considered the possibility that self-pollinated B_III_ embryos (self-pollinated apomeiotic embryo sacs; e.g., AAB → AABB and AAA → AAAA) could aggregate in Q2 as well. Only conducting FCSS and sequencing on the same seed could indisputably distinguish apomixis from B_III_ selfing and B_III_ selfing from B_III_ outcrossing. However, in the present study, individual seeds were destructively sampled for one method or the other. Nevertheless, since individuals with putative B_III_ embryos (detected by FCSS) commonly displayed some level of apomixis, we concluded self‑pollinated B_III_ embryos were unlikely to lead to false positives in identifying facultative apomicts. Q3 represented sexual outcrossing, including gamete reduction, recombination, segregation, and foreign pollination or xenogamy. Lastly, Q4 was predicted to represent two scenarios: 1) all phenomena listed for Q3, although more specifically involving the fusion of gametes containing fixed haploblocks among select mating partners or 2), the foreign pollination of an apomeiotic egg cell (B_III_ outcross). Genotypes were determined to be facultative apomicts if at least one embryo fell in Q2.

To evaluate how our models performed with fewer compared sites (mainly a consequence of low sequencing coverage), we subsampled sites from 3 embryos representing 20 maternal genotypes that each had ≥ 5000 compared sites in their complete datasets (S4 Table). Pruned embryo VCFs were subsampled for 150, 500, 1000, and 2000 random sites and assigned a coverage value based on the median coverage for all embryos with ± 100 sites (0.3X, 0.5X, 0.6X, and 0.9X coverage, respectively). For the 150 and 500 site comparisons, 50/60 of the embryos were assigned the same reproductive quadrant as their full datasets. For the 1000 and 2000 site comparisons, 53/60 and 56/60 were assigned identical quadrants, respectively. Most mismatches within each subsampling level represented shifts in sexual classifications (Q1, Q3, and Q4) rather than shifts in asexuality to sexuality and vice versa (Q2 → other or other → Q2). For the latter scenarios specifically, the 150, 500, 1000, and 2000 had four, four, two, and zero embryos involving Q2 misclassifications. Taken together, these results demonstrate accuracies of 83.3 – 93.3% across all sequencing quadrants even when comparing very few sites, and higher accuracies (93.3 – 100%) when simply distinguishing clonally- versus sexually-produced embryos.

### Screening-by-sequencing results of diverse *Malus* species

**Fig 3:**
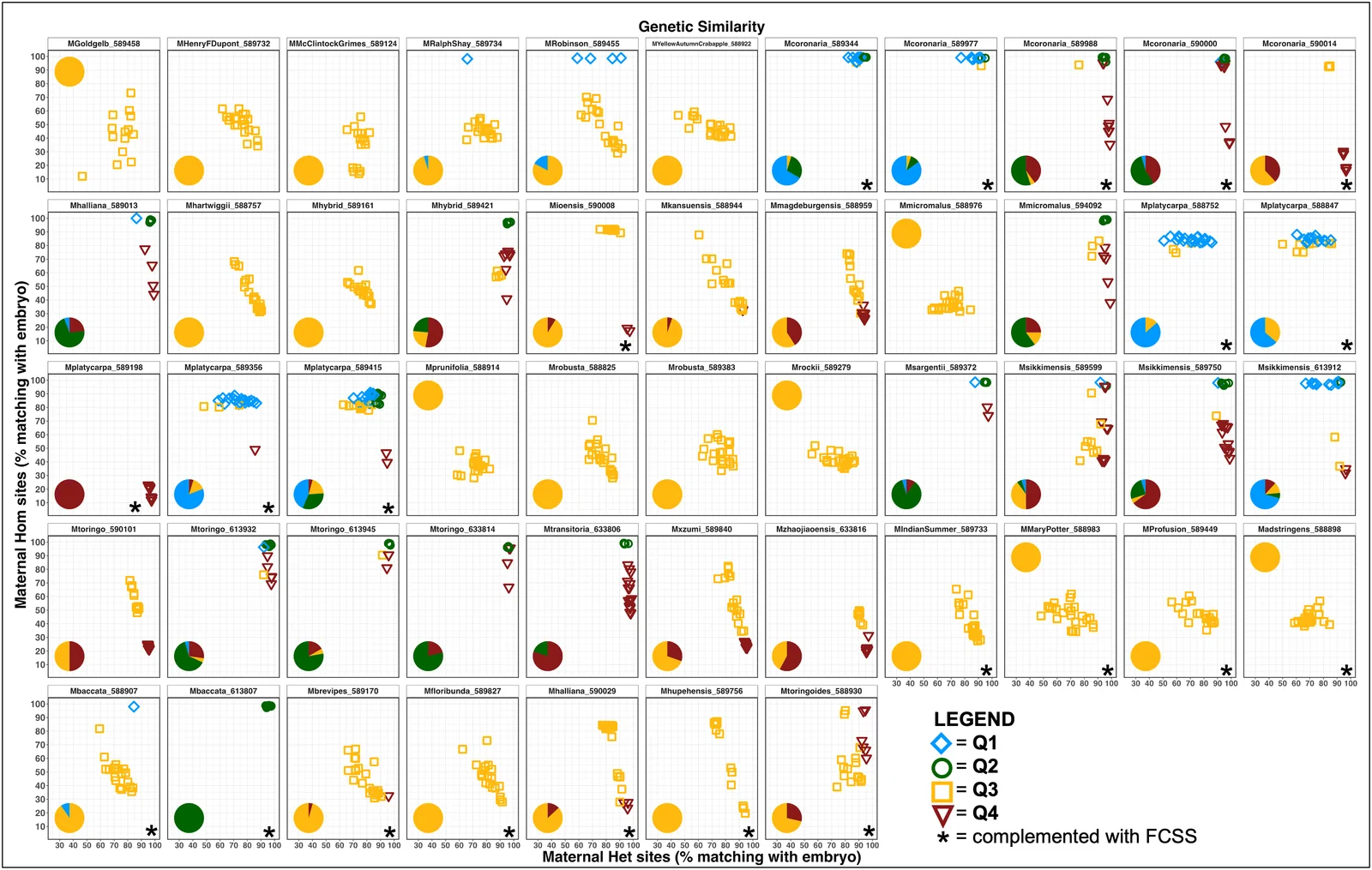
Low-pass sequencing screen results for 51 *Malus* genotypes maintained by the USDA in Geneva, NY. The layout of the subplots are identical to those in Fig 2A, with added (*) indicating which genotypes were also complemented with the flow cytometry seed screen. Further details can be found in S2 and S3 Tables.

The low-pass screening-by-sequencing results for 51 *Malus* genotypes maintained by the USDA in Geneva, NY are shown in Fig 3 (also see S2 Table). Most genotypes (n = 34) produced embryos only through putative sexual processes according to our quantile regression models (Q1, Q3, and Q4). In accordance with *Malus* species mainly reproducing through obligate outcrossing, the majority of embryos from these genotypes fell in Q3. Still, rare selfing events do occur in *Malus* and polyploidy may break down self-incompatibility [63]. In concordance with past literature, a few selfing events (Q1) were detected in diploids (*M. baccata* 588907, *M.* ‘Robinson’ 589455, *M.* ‘Ralph Shay’ 589734), but most cases of autogamy were found in polyploids, and more specifically in tetraploids. All obligate outcrossing individuals are diploids except for *M. ×* *domestica ‘*Goldgelb 55544’ 589458 and *M. ×* *domestica* ‘McClintock Grimes’ 589124, which are both triploids. These findings are consistent with other observations that the cultivated apple, irrespective of ploidy, reproduces through sexual processes [59,60].

The low-pass sequencing data identified 17 previously-unknown facultative apomicts. Most facultative apomicts screened here are polyploids (triploid or tetraploid), a common feature of gametophytic apomicts [58]. An exception was *M.* hybrid (*M. rockii*) 589421, which is recorded as diploid in the GRIN-Global database [62]. Overall, most apomictic accessions confirmed here belong to species native to eastern Asia and North America, with previous documentation of apomixis in their populations, including *M. hupehensis*, *M. sargentii*, *M. baccata*, *M. sikkimensis*, *M. toringo*, *M. transitoria*, *M. platycarpa*, and *M. coronaria* [4,53,56,64]. However, it is worth noting species classifications did not perfectly correspond to reproductive mode, highlighting the need to determine apomixis at the individual level. For example, triploid *M. baccata* 613807 reproduced through facultative apomixis according to the sequencing screen while diploid *M. baccata* 588907 was shown to only produce embryos sexually (Fig 3). Most *M. toringo* accessions screened were shown to be facultative apomicts as has been recorded elsewhere in the literature [56], although diploid *M. toringo* 590101 reproduced embryos only through sexual processes.

**Fig 4:**
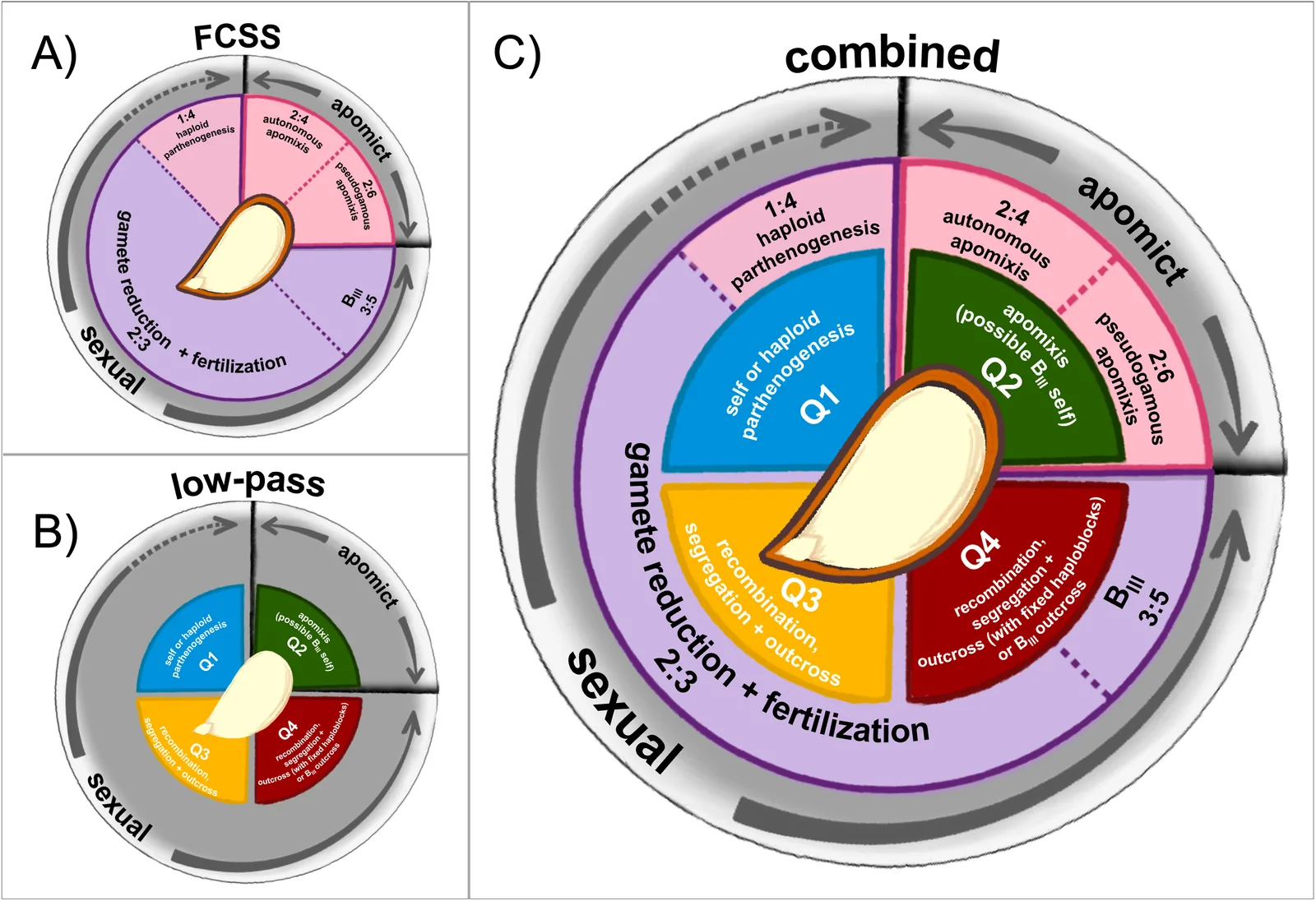
An illustration comparing and contrasting the types of conclusions that can be drawn from A) FCSS and B) low-pass sequencing alone and when C) combined. **A)** The FCSS method measures relative DNA amounts between endosperm and embryo tissues and deduces reproductive outcomes based on this ratio. In addition to predicting gamete reduction and fertilization, it has the capacity to distinguish autonomous versus pseudogamous forms of apomixis and capture genome increases or decreases across generations (B_III_ or haploid parthenogenesis, respectively). The reproductive scenarios are labeled with examples of an embryo: endosperm DNA ratio for that scenario given both parents are diploids. In the present study, we considered any form of genetic change from mother to child a form of sexual reproduction (i.e., involving gamete reduction, recombination, segregation, or fertilization), and the outer circle labels and coloration are meant to reflect this. However, as haploid parthenogenesis does not involve syngamy of the egg and sperm, in some contexts this scenario is considered a form of apomixis. The partially dotted outer arrow and pink shading for that sector depict this nuance. **B)** In contrast with FCSS, sequencing-based approaches directly test the genetics of the embryo and can distinguish clonal progeny from other sexual scenarios involving the addition of new alleles or segregation of loci. While sequencing based approaches for determining ploidy is an area of active research, it especially remains a challenge for low-coverage datasets. Note that B_III_ selfing is included in the Q2 quadrant as a hypothetical, as the frequency of these events is unknown and could not be estimated from the present data. **C)** Summarizes the overlap in these methods and illustrates the resolution both types of data provide together. For example, a true clonal embryo in sequencing quadrant Q2 would be expected to show embryo: endosperm ratios of 2: 4 or 2: 6. A seed with FCSS ratio 2: 3 would be expected to show genetic differences reflecting scenarios in Q1, Q3, or Q4.

According to the low-pass sequencing screen, triploid apomicts *M. sargentii* 589400 and *M. hupehensis* 633818 (Fig 2A), *M. transitoria* 633806, *M. halliana* 589013, *M. micromalus* 594092, *M. sikkimensis* 589750, *M. platycarpa* 589198, *M. coronaria* 590000, *M. coronaria* 589988, *M. toringo* 633814, *M. toringo* 613945, and *M. toringo* 613932 frequently produced embryos in Q4 (Fig 4). Available FCSS data from *M. sargentii* 589400, *M. platycarpa* 589198, *M. coronaria* 590000, and *M. coronaria* 589988 confirmed pseudogamous apomixis and genome increase as predominant modes of reproduction, supporting the prediction that Q4 includes embryos produced through apomeiosis and foreign pollination (B_III_ hybridization or outcross). Still, complementary FCSS observations in species producing only sexual seeds with canonical 2: 3 embryo: endosperm ploidy ratios (*M. hupehensis* 589756, *M. halliana* 590029, *M. ioensis* 590008, and *M. coronaria* 590014), also produced embryos in Q4. These observations strongly imply Q4 also represents other sexual situations, likely those involving the segregation of fixed alleles or large haplotype blocks among preferred mating partners as postulated above. In contrast to triploid apomicts, tetraploid apomicts (*M. sikkimensis* 589599, *M. sikkimensis* 613912, *M. coronaria* 589344, *M. coronaria* 589977, *M. sargentii* 589372, and tetraploid *M. platycarpa* individuals) more regularly produced selfed embryos (Q1), which may be an indirect consequence of severe pollen sterility in triploid apples.

### Discrepancies between low-pass sequencing and FCSS results

Low-pass sequencing and FCSS generally agreed with one another when identifying progeny produced through sexual or apomictic processes, albeit with some noteworthy discrepancies. There were several genotypes in which FCSS captured at least one apomictic seed while the sequencing screen predicted all embryos to be sexually-derived. For instance, 5 of the 25 seeds analyzed by FCSS for tetraploid *M. toringoides* 588930 were presumably products of apomixis (Table 1). Two Q4 embryos for this genotype failed the homoallelic threshold of 95.8% similarity by ≤ 1 percentage point – which taken together with the FCSS data, raises suspicion they may be false negatives (Fig 3). Depending on the application, these edge cases may deserve special attention given the models’ expected false negative rate of 5%.

All four tetraploid *M. platycarpa* genotypes (588752, 588847, 589356, and 589415) were facultative apomicts according to the FCSS, producing apomictic seed at variable proportions (Table 1). However, the sequencing screen only captured apomictic embryos for *M. platycarpa* 589415, which also had the highest rate of apomixis according to the FCSS data (Fig 3 and Table 1). Reducing the heteroallelic boundary for these genotypes by 1.7 percentage points was sufficient to reassign one Q1 embryo from 588752, 588847, 589356 to Q2. Therefore, we hypothesize the possible false negatives in the sequencing data for tetraploid *M. platycarpa* genotypes are most likely due to their models’ failure to account for all sources of error. Given heteroallelic sites were more susceptible to biological noise in the model constructed for all other genotypes, it is believed the biological penalty for tetraploid *M. platycarpa* embryos should be harsher.

On the contrary, FCSS data occasionally captured putative apomictic seeds in diploid genotypes where the sequencing data indicated these individuals do not produce clonal embryos (*M. asiatica* 594099, *M.* ‘Profusion’ 589449, *M. brevipes* 589170, and *M. ×* *adstringens* 588898). While sampling variability cannot be ruled out, these observations are suspected false positives in the FCSS data as all sequenced embryos from these genotypes were substantially removed from Q2 boundaries.

Finally, one triploid genotype (*M. platycarpa* 589198) only produced Q4 embryos according to those sequenced in 2023, while FCSS results from 2024 seeds indicated both pseudogamous apomixis and genome increase (fertilization of an unreduced egg cell) as its primary modes of reproduction. As *M. platycarpa* 589198 did not show the same spike-in trends as its tetraploid counterparts and all Q4 embryos showed considerable genetic distances from the homoallelic clonal boundary, it is hypothesized these inconsistencies are not due to technical limitations of either method. Instead, they may simply be due to chance sampling, or spring conditions differing between years may have altered the reproductive rates of apomixis and genome increase in this individual since apomixis is known to be influenced by environmental factors such as temperature [25,26,28].

## Discussion

In the present study, we took advantage of the immense genetic resources maintained by the USDA to develop a low-pass, whole genome sequencing approach to detect apomixis (clonal seed production) in *Malus* genotypes. A total of 55 individuals representing over 20 recognized species, hybrids, and cultivated apples were screened by sequencing embryos at a range of WGS coverage (Fig 1) and comparing the maternal parent and progeny sequence data with open-source bioinformatic tools. A subset of these individuals (n = 26) was also analyzed by FCSS to validate and complement the low-pass approach, and both types of data were largely consistent with one another with some exceptions that partly illustrate each method’s strengths and weaknesses (Fig 4). While previous studies have used molecular approaches to detect clonal reproduction in populations, our study is the first to do so without pre-designed molecular markers and to show that low-pass WGS is a robust option to screen for clonality and other forms of reproduction.

We used plate-based DNA extractions and Twist Bioscience’s 96-plex library kit to create libraries for over 1,200 open-pollinated embryos. Importantly, *Malus* genomes are small (650 Mb), so the combined costs of library preparation (using half-volume reactions) and sequencing each embryo to 3X coverage came to $10.50 per sample. The relationship between the number of sites analyzed and sequencing coverage indicates that reliable conclusions, with hundreds to thousands of biallelic sites still being captured for comparison, can be drawn at 1.5X coverage even for tetraploids (Fig 1B). Our subsampling experiments also indicate high accuracies with relatively few sites (and by proxy, lower coverage). However, to minimize sample dropout at lower sequencing coverage, refinements in kit chemistry and protocol (e.g., autonormalization by ligation prior to library preparation) would be necessary to significantly narrow the sample coverage distributions around the mean (Figs 1A and 1C).

Our study also demonstrated the importance of using biological and technical controls to model error rates in low coverage sequencing data and to identify abnormal samples. Based on our results, the larger error seen only in the tetraploid *M. platycarpa* samples may be an intrinsic feature of the species at this ploidy level. For instance, a compound produced by these individuals may interfere with the fidelity of the DNA polymerase during PCR-based library preparation, introducing random errors. This is also consistent with the fact that tetraploid *M. platycarpa* genotypes had higher error rates at homoallelic sites as compared to heteroallelic ones. The failure to classify apomictic embryos on the cusp of heteroallelic boundaries could also indicate higher amounts of biological noise specific to these genotypes. Complementary methods such as FCSS, which suggested all four individuals produced clonal progeny (Table 1), may be required to resolve such special cases.

A simple workflow diagram illustrating the low-pass method is presented in S4 Fig. This method was explored in *Malus* given the availability of germplasm and its amenability to FCSS as a supporting method, but it could be adapted to other species as well. The ideal strategy for determining reproductive mode will ultimately depend on a species’ biology and any technical hurdles that exist for the researcher, such as affordable access to a flow cytometer versus a sequencing core. Ploidy, genome size, feasibility for FCSS, reference genome availability, breeding tendencies, allele frequencies, and heterozygosity levels should all be considered when designing low-pass screening-by-sequencing experiments. For instance, heterozygous outcrossing species (including *Malus*) would be expected to require less sequencing coverage for robust conclusions compared to highly inbred populations, as more informative sites would be captured by chance. A small pilot experiment including several genotypes representing the range of diversity for a particular system may be necessary before expanding the scale of screening. For larger genomes, imputation may assist with data sparseness [65], or a custom probe set coupled with enrichment sequencing would ensure adequate coverage of polymorphic locations that are evenly distributed across the genome; however, this approach would require the design of species- or genera-specific markers [66]. Nevertheless, we expect declining costs, improvements in sequencing chemistries, and DNA extraction automation to regularize sequencing-based approaches for investigating reproductive plant biology.

In combination with the FCSS data, the sequence data created a highly resolved reproductive profile for an individual. The types of reproduction each method is able to distinguish, as well as how they complement one another, is summarized in Fig 4. Sequencing offers the most direct approach to identifying clonal and sexual progeny and may in fact be the only suitable option to screen for reproduction when FCSS is not feasible. FCSS is currently the best technique to capture changes in ploidy in the next generation as long as the maternal genotype’s ploidy is known. Predicting ploidy based on various types of sequence data is actively being investigated, but model selection can vary depending on the biology of a species and greater sequencing coverages beyond those generated here are recommended for high accuracy [67].

Sequence-based screening also resolves certain debatable interpretations of FCSS (e.g., autonomous apomixis vs. embryo priming) [43]. In the present study, putative apomixis events were detected in certain diploid individuals, including *M. ×* *adstringens* 588898, *M. brevipes* 589170, *M.* ‘Profusion’ 589449, and *M. asiatica* 594099, yet all embryos examined by the sequencing screen reflected recombination, segregation, and cross pollination (Q3 and Q4; Table 1 and Figs 2 and 3). Many plant species including *Malus* are known to produce occasional unreduced gametes through meiotic abnormalities such as first or second division restitution, so it is possible these events were not actually clonal [37,60]. Applying FCSS and genotyping methods to the same seed would resolve these questions, as has been recently done for *Rubus* [68]. Alternatively, classical developmental staging using confocal microscopy and advanced imaging technologies would be informative on the mechanism, as was elegantly shown in several Asteraceae taxa [69]. In summary, emerging multi‑faceted approaches promise to resolve the environmental and genetic influences on plant reproduction with unmatched precision.

In addition to directly testing the genetics of the progeny, another strength of a low-cost sequencing method to study reproduction is that it easily discerns self-fertilization (autogamy) from outcrossing events (xenogamy), which provides information on the breeding dynamics of specific individuals and populations. For instance, the distributions of embryos’ genetic similarities relative to their maternal parent in Fig 3 highlights questions regarding *Malus* mating systems, including those on pollen competition and interspecific barriers. The detection of rare selfing events in *Malus* implies that even if individuals are self-compatible, xenogamy typically prevails over autogamy. Although this phenomenon has been widely documented, the underlying genetic and environmental causes are complex and vary between species [70].

Our experimental conditions included a large orchard of *Malus* species native to regions worldwide, which likely increased mating among individuals that would not normally encounter one another in nature. Still, despite this fact, certain North American sexuals (*M. ioensis* 590008, *M. coronaria* 590014) and facultative apomicts (polyploid *M. platycarpa* and *M. coronaria* individuals) show highly constrained genetic distributions relative to their maternal parent that imply preferential mating. Tetraploid North American species (*M. platycarpa* 589415, 588752, 588847, and 589356; and *M. coronaria* 589344 and 589977) deviate mainly on the x-axis, indicating apomixis and/ or selfing to be their primary modes of reproduction despite the abundance of intra- and interspecies partners at a variety of ploidy levels. The triploid accessions of *M. coronaria* (590000 and 589988) and *M. platycarpa* (589198) reproduced mainly through genome increases (B_III_ hybridization) and apomixis. Although it is possible the abundance of interspecific pollen increased the prevalence of asexuality in some of these individuals, a recent study did not find an association between pollen donor of the endosperm (interspecific or heterospecific vs intraspecific or conspecific) and asexual embryo formation in open-pollinated *M. coronaria* growing among feral *M. ×* *domestica* [71]. Taken together with previous findings that have identified low genetic diversity in certain populations of *M. coronaria* [72,73], these results have broader implications for species conservation, especially as it applies to the evolution of reproductive systems in populations with variable ploidy levels and high rates of selfing [73,74].

*Malus* is a taxonomically challenging genus of over 50 named species with reticulate evolutionary processes such as hybridization and polyploidy in addition to apomixis [61,75]. Gametophytic apomixis in *Malus* is dominant and the genetic components regulating apomeiosis and parthenogenesis are thought to act separately, which has also been shown in *Taraxacum*, *Cenchrus,* and *Hieracium* [4,11,12,25,76]. The prevailing question remains as to whether the genes controlling these processes in *Malus* are physically linked together in chromosomal space, although the FCSS results provide indirect insight on this topic. In the facultative apomicts confirmed in the current work, haploid parthenogenesis and B_III_ embryos (presumably from the pollination of an apomeiotic egg cell) always co-occurred with some level of pure apomixis, implying apomictic components may be commonly inherited together. The sequence data generated here represents some of the most diverse WGS resources for apomictic individuals to date and can be used in future comparative -omics studies to map apomixis genes and describe their evolutionary trajectory in apple. These genes have enormous potential for plant breeding, and characterizing them across diverse angiosperm lineages could mitigate existing obstacles for engineering apomixis in crop species [17,21].

## Materials & methods

### Plant material

All plant materials (leaves and seed) were obtained from the National Plant Germplasm System (NPGS) *Malus* collection, which is maintained by the United States Department of Agriculture (USDA) at the ARS Plant Genetic Resources Unit in Geneva, New York [77]. Accessions were chosen for screening based on previous accounts of apomixis in *Malus*. Specifically, we selected individuals of species that met any of the following criteria:

1) they had any documentation of producing apomictic seed in the literature (e.g., *M. hupehensis, M. sargentii, M. sikkimensis, M. toringo, M. toringoides, M. baccata, M. platycarpa,* and *M. coronaria*), 2) were hybrids with documentation of apomixis (e.g., *M.* ‘Indian Summer’) or had a potential apomictic species in their pedigree (e.g., *M.* ‘Mary Potter’, *M.* ‘Yellow Autumn Crabapple’), 3) were triploid accessions of *M. ×* *domestica* (e.g., cultivars ‘McClintock Grimes’ and ‘Goldgelb 55544’), or 4) were wild diploids with no known apomixis (e.g., *M. floribunda*, and *M. ioensis*). The ploidy level of each maternal genotype was determined previously by NPGS with flow cytometry and is listed with other characteristics of the accession in the GRIN-Global database [62]. Information on which accessions were included in the present study, including the number of embryos sequenced and whether there is complimentary FCSS data in the present work, is given in S1 Table.

Open-pollinated seeds were isolated from randomly-collected fruits in the fall of 2023 and 2024, then washed in a 30% commercial bleach solution with 0.02% Triton X-100 and rinsed with tap water. They were then stored at 4 °C with drierite until dissection for DNA extraction or for FCSS. Embryo tissue was used for DNA extraction to avoid the need for seed stratification and planting, and because it was easily isolated from the seed coat and diminutive endosperm using a Motic K-400 Stereo Microscope. Individual embryos were placed in wells of a 96-deepwell plate (Qiagen, USA, Valencia, CA; cat:19560) on dry ice and stored at -80 °C until DNA extraction. Leaves for DNA isolation of the maternal genotype were either 1) collected in the fall of 2023, lyophilized, and stored dry at room temperature; or 2), were collected fresh in the spring of 2024, flash frozen in liquid nitrogen, and stored at -80 °C until DNA extraction.

### DNA extraction

Up to 96 embryo DNAs were extracted at a time using a modified CTAB protocol [78]. Briefly, tissue was ground frozen with 4 mm glass beads using a SPEX SamplePrep 1600 MiniG (Cole-Parmer sample prep, Metuchen, NJ), and 500 μL of warm lysis buffer was added directly to the sample and vortexed vigorously. The homogenate was incubated at 55 °C for at least an hour on a plate incubator (Benchmark Scientific Inc., USA, Edison, NJ; model: H6004) set to at least 1000 rpm. Next, 500 μL of 1:1 phenol: chloroform was added, mixed by inversion, then centrifuged at max speed (3486 rcf) in an Eppendorf 5810R bucket centrifuge with attachment A-2-DWP-AT (Eppendorf, Hamburg, Germany). Then 500 μL of a 24:1 chloroform: isoamyl alcohol solution was added to the recovered supernatant, and mixed by inversion before another centrifugation step and supernatant recovery. An equal volume of ice-cold isopropanol was added to the recovered supernatant, mixed by inversion, and the DNA was allowed to precipitate overnight in a -20 °C freezer. The following day, DNA was pelleted by centrifugation at max speed for 20 minutes, washed twice with 70–80% ethanol, and eluted in 0.1X TE buffer. DNA quality and concentrations were assessed with a NanoDrop Eight spectrophotometer (ThermoFisher Scientific, Waltham, MA) and a DeNovix model DS-11 FX (DeNovix Inc., Wilmington, DE) with an Invitrogen 1X dsDNA BR kit (ThermoFisher Scientific, Waltham, MA; cat: Q33266), respectively. Most maternal genotype DNAs were extracted in parallel in a separate well alongside their embryos; however, in some cases, maternal DNA was extracted separately using the DNEasy Plant Pro kit (Qiagen USA, Valencia, CA; cat: 69204).

### Library preparation & sequencing

Libraries for the 55 maternal genotypes were prepared as single reactions using either the SeqWell PurePlex High Complexity (Alpha) DNA library prep kit (SeqWell, USA, Beverly, MA; cat: 301400) or the NEBNext Ultra II FS DNA library prep kit for Illumina (New England Biolabs, USA, Ipswich, MA; cat: E7805S) according to the manufacturer’s instructions. DNA inputs ranged between 80ng - 430ng. Libraries for 1,216 embryos were prepped in pools of up to 96 samples according to the Twist Bioscience 96-plex library prep kit instructions but in half volume reactions (Twist Bioscience, USA, South San Francisco, CA; part:106543). Between 18 and 24 embryos were sequenced per maternal parent. To better control for technical error rates associated with particular genotypes (i.e., tetraploid *M. platycarpa*), library differences, and lower sequencing coverages, 1 – 3 maternal DNA extracts were also prepared alongside embryos sequenced to 3X target coverage (spike-ins). Samples were normalized prior to library preparation so that DNA inputs ranged between 10 ng - 40 ng per sample depending on the plate.

All samples were sequenced on a NovaSeq X plus with 10B flow cells in the 150 bp paired-end format at Discovery Life Sciences (Huntsville, AL). Maternal genotypes were pooled in equal concentrations with 16–19 individuals per lane, bringing the target coverage for each maternal parent to 28X - 33X assuming a monoploid *Malus* genome size of approximately 650 Mb. Whole genome target coverages of 6X and 3X for embryo samples were attained by sequencing 96 or 192 samples on a sequencing lane, respectively. Plates of embryos/ spike-ins were demultiplexed off the sequencer according to their i7 adapter sequence, and fgbio version 2.2.1 was used to demultiplex individual samples based on their i5 sequence [79].

### Optimizing the variant calling pipeline

Given potential sources of noise in low-pass sequencing datasets, variant calling error rates were first optimized by downsampling all 55 deeply-sequenced maternal libraries and comparing calls in these subsets to the full dataset. Maternal parent libraries were downsampled using seqtk (https://github.com/lh3/seqtk) at coverage levels representative of their embryo populations (2X, 4X, and 12X for maternal parents with 6X coverage embryos and 1X and 3X for those with 3X coverage embryos). The noise-to-signal ratio was improved by iteratively testing different variant quality filters, noting the number of mismatches between subsets and the full maternal dataset, exploring the reasons for mismatches through manual examination of alignments in IGV version 2.13.0 [80], and adjusting the filters accordingly. When optimizing filters, heteroallelic (0/1) site error and homoallelic (0/0 or 1/1) site error were examined separately. Generally, subsets with lower coverage had higher variability in error rate, but the overall accuracy rate was > 95% in nearly all cases (S1 Fig).

Fastp version 0.23.4 was used to trim and remove sequence adapters, discard reads with > 30% low-quality bases (q < 15), and remove PCR duplicates from the demultiplexed, compressed raw fastq files [81]. Reads for all samples were aligned with bwa-mem version 0.7.17 to haplome A of the *Malus ×* *domestica* ‘Honeycrisp’ v1.1.a1 genome, which was downloaded from the Genome Database for Rosaceae [82–84]. Samtools version 1.19.2 was used to extract raw and filtered read alignments and calculate average coverage, as well as sort and filter the alignments [85]. Raw average coverage was estimated for each sample by multiplying the number of aligned reads prior to quality filtering by 150 (the maximum read length) and dividing it by the monoploid apple genome size in base pairs (650,000,000). Only alignments with mapQ > 30 were used for variant calling, and secondary alignments (multi-mapping reads) were excluded.

Both bcftools (v1.19) and freebayes (v1.3.1) were used to call genotypes to enhance accuracy [85,86]. Each sample was called and filtered separately, and only biallelic SNPs were considered. Low (LDP) and high depth (HDP) filters, or the minimum and maximum number of reads supporting a site, were set according to the ploidy of the maternal parent and the embryo’s approximate coverage. For embryos of diploid, triploid, and tetraploid parents, a minimum of 10, 11, and 12 reads, respectively, were required to support a genotype call. For embryos with average coverages of 1X, 2X, 3X - 6X, and> 6X, the maximum number of reads allowed for any given call was 25, 30, 30, and 40, respectively. Spike-ins were processed in the same manner as embryos. Maternal genotypes’ LDP cutoff was set to 15, and the HDP was set to five times the filtered average coverage. In addition to the depth filters, only biallelic sites with genotype qualities (GQ) greater than 30 and site qualities (QUAL) greater than 50 were kept for downstream analysis for embryo samples. Sites that were homozygous reference with QUAL > 50 were also kept in the maternal parent.

After calling and filtering, the SelectVariants –concordance function from GATK version 4.6.0.0 was used to keep sites that were present in both the bcftools and freebayes VCF files [87]. Concordant embryo files were pruned with bcftools +prune, selecting only one site per 1000 bp to reduce bias in the results due to linkage. A limitation of the pruning function allowed only the preservation of variant sites relative to the reference genome (0/1 or 1/1 locations), but our controls demonstrated this did not affect our ability to detect clonal embryos. Afterwards, the concordant, filtered, maternal VCF was merged with its concordant, pruned embryo VCFs. Bcftools gtcheck was used to determine the number of mismatching sites between maternal parent and each embryo [85].

### Statistical determination of clonal boundary cutoffs with biological and technical controls (spike-ins)

Known obligate sexually-reproducing genotypes (*M. spectabilis* 588917 and *M. asiatica* 594099) and facultatively apomictic genotypes with high penetrance (*M. hupehensis* 633818 and *M. sargentii* 589400) were included in the initial 6X coverage experiments to distinguish true clonal embryos from those produced through sexual and intermediate processes. High-penetrant apomicts were defined here as producing > 50% clonal progeny. Embryos clustering tightly near 100% similarity on both axes in *M. hupehensis* 633818 (n = 19) and *M. sargentii* 589400 (n = 14) were considered true biological clones representing apomixis, or the absence of gamete reduction, recombination, segregation, and cross fertilization. This excluded three embryos slightly deviating from the main cluster in *M. hupehensis* 633818 (Fig 2A). These data were used to understand error rates associated with biological noise, and the coverage of these embryos ranged from 2.6X – 12.9X.

Maternal spike-ins for 40 genotypes were prepared alongside their embryos to correct for technical error rates (e.g., potential bias between the single SeqWell or NEBNext library preps and Twist Bioscience’s 96-plex kit) and to resolve the discrepancies initially seen in the *M. platycarpa* 589415 data. The spike-ins from tetraploid *M. platycarpa* accessions (*M. platycarpa* 589415, 588847, 589356, and 588752) were excluded from the dataset used to define clonal boundary cutoffs for all other genotypes. Only spike-ins with at least 50 hetero- and 50 homoallelic sites for comparison were used in statistical analyses. In total, 77 spike-ins from 34 maternal genotypes, ranging in coverage from 0.4X – 8.4X, were used to model clonal boundary cutoffs.

Data were imported into R version 4.6.0 [88]. Tidyverse, ggplot2, quantreg, car and lme4 packages were used for data reformatting, generating plots, and performing statistical analyses [88–93]. Heteroallelic and homoallelic boundaries for clonal embryos were modeled separately. Quantile regression was used to address deviations from normality and heteroskedasticity. An analysis of covariance (ANCOVA) of two quantile regression models (tau = 0.05) including and excluding the coverage × experiment interaction term indicated the biological controls’ and spike-ins’ error rates responded similarly to changes in coverage for both heteroallelic and homoallelic sites (p = 0.49). Therefore, biological controls and spike-ins were pooled to increase statistical power for modeling. We proceeded to use quantile regression to model the heteroallelic clonal boundary cutoff with a 5% false negative rate using average coverage and experiment type (biological or spike-in) as covariates. The intercept of the biological controls served as the baseline error rate for heteroallelic sites, and the final heteroallelic boundary was adjusted according to the embryo’s sequencing coverage. Another series of ANCOVA tests for the homoallelic clonal boundary cutoff indicated coverage and experiment type did not significantly affect error rates. Thus, an intercept-only quantile regression model was fitted for homoallelic sites. This intercept was used as the homoallelic boundary for all embryos regardless of their coverage (= 95.8%), excluding those produced by tetraploid *M. platycarpa* genotypes.

Separate statistical models and clonal boundaries were established for embryos derived from the four tetraploid *M. platycarpa* genotypes using these genotypes’ spike-ins. Due to their limited sample size (n = 8), formal ANCOVA testing of the coverage × experiment type at the 5^th^ percentile was not feasible. However, visual inspection of the data suggested similar coverage-response slopes for the tetraploid *M. platycarpa* spike-ins, normal spike-ins, and the biological controls. We therefore proceeded with quantile regression models (tau = 0.05) for the tetraploid *M. platycarpa* spike-ins, with heteroallelic sites being modeled as a function of coverage and homoallelic sites being modeled simply as a function of the intercept. Since these samples had higher error rates than the biological controls, the previously-determined intercept of the biological controls could not be directly used as a baseline error rate for heteroallelic sites of tetraploid *M. platycarpa* embryos. Instead, an additional ‘biological penalty’ for heteroallelic sites was calculated to help address potential biological sources of noise in the tetraploid *M. platycarpa* genotypes’ embryos. This penalty for heteroallelic sites was calculated as:


$$\begin{array}{c} Biological\;Penalty\;=\;Het\;predicted\;\text{5}th\;percentile(normal\;spike-ins)\;-\;Het\;predicted\;\text{5}th\;percentile(biological\;controls) \end{array}$$

Where the het predicted 5^th^ percentiles were the values predicted by the heteroallelic quantile regression model for each respective experiment type. Whether an embryo passed its heteroallelic and/ or homoallelic clonal boundary cutoff predicted the reproductive scenario it was derived from as described in the text. Bcftools and basic command line functions were used to subsample sites within the pruned embryo VCFs to assess consistency of these models [85]. The subsampled VCFs were then compared to their maternal parent in an identical fashion as the full embryo datasets.

### Nuclei isolation and preparation for FCSS

668 seeds of 12 *Malus* species and several cultivated hybrids (totaling 26 individuals; S3 Table) were first prepared from pomes and cut in half. Only well-developed seeds were used for the detection of reproductive modes based on a FCSS [34]. Each sample consisted of one half of a seed with the internal standard (*Carex acutiformis* 2C = 0.82 pg; [94]). DAPI (4’,6-diamidino-2-phenylindole) staining was used with a Partec CyFlow ML cytometer equipped with a 365-nm UV LED. A simplified two-step protocol was followed [95]. A slight modification consisting of using a larger amount of the Otto I buffer (0.7 ml; according to [96]) and shortening of incubation time (max 2 min) was adopted. The ploidy of the embryo and the endosperm was calculated from the peaks of the fluorescence histograms, which are given in S3 Table.

## Supporting information

S1 Fig Variant calling accuracy rates (% of sites matching) between a downsampled maternal library and the full maternal library. Number of downsampled libraries in each coverage bin are given above the boxplots, while the mean number of sites compared after filtering is given below each boxplot. Subsets were filtered to compare only those with at least 10 heteroallelic and 10 homoallelic sites.
(TIFF)

S2 Fig Locations of biallelic sites compared between a maternal genotype and two embryos each from *M. spectabilis* 588917 (pink) and *M. hupehensis* 633818 (brown). The distributions shown are for **A)** embryos of the lowest coverage for each genotype and **B)** an embryo with an average coverage for that genotype.
(TIFF)

S3 Fig Histograms showing the % similarities for each site type between maternal leaf DNA extracts prepared with the SeqWell or NEBNext library kits and the Twist Bioscience’s 96-plex kit (spike-ins). Spike-ins prepared from tetraploid *Malus platycarpa* genotypes all showed substantially lower similarities to the same DNA prepared with SeqWell or NEBNext. These observations are indicated with an *.
(TIFF)

S4 Fig A schematic illustrating the main steps involved in the low-pass screening method for apomixis and other forms of reproduction. Depending on the application, the maternal parent may be prepared separately and sequenced to higher coverage to capture more sites that will be sparsely distributed in the low-pass sequenced embryos, or multiple maternal spike-in replicates could be pooled after sequencing to represent the full maternal dataset depending on desired coverage. Biological controls, especially representing clonal reproduction, should be incorporated in the design for later statistical modeling of the clonal boundaries. Coverage per sample is adjusted by the number of samples pooled on a sequencing lane. Data processing may be streamlined with just a handful of open-source tools, and statistical modeling and visualization is completed in RStudio.
(TIFF)

S1 Table List of accessions’ taxonomic classification as stated in the USDA germplasm database (August 2025), their PI numbers, ploidy, the brand of library kit used, the number of embryos sequenced, and the number of seeds analyzed for flow cytometry. Fruiting years the seed was collected in are in parentheses. The embryos derived from genotypes highlighted in blue were sequenced at a target coverage of 6X for their 2023 seed (up to 96 samples per sequencing lane). All other embryos were sequenced at a target coverage of 3X (up to 192 samples per sequencing lane), with most being sequenced in fruiting year 2024.
(XLSX)

S2 Table Sequencing comparison results between maternal parents and 1,158 embryos (58 embryos failed the 50 heteroallelic and 50 homoallelic site minimum filter). **mother** = the maternal parent an embryo is derived from; **embryo** = sample ID for the sequenced embryo; **het_sites_total** = total number of sites compared between an embryo and its maternal parent at sites where the maternal parent was heteroallelic (0/1); **het_sites_mismatch** = number of sites mismatching between an embryo and its maternal parent at sites where the maternal parent was heteroallelic (0/1); **het_match_percentage** = percent of sites matching between an embryo and maternal parent at sites where the maternal parent was heteroallelic (0/1); **hom_sites_total** = total number of sites compared between an embryo and its maternal parent at sites where the maternal parent was homoallelic (0/0 or 1/1); **hom_sites_mismatch** = number of sites mismatching between an embryo and its maternal parent at sites where the maternal parent was homoallelic (0/0 or 1/1); **hom_match_percentage** = percent of sites matching between an embryo and maternal parent at sites where the maternal parent was homoallelic (0/0 or 1/1); **total_sites** = total number of sites compared between embryo and its maternal parent; **total_mismatch** = total number of sites mismatching between an embryo and its maternal parent; **total_match_percentage** = percent of all sites matching between embryo and maternal parent; **target_cov** = the target coverage for that embryo; **seed_year** = fruiting year the seed was collected; **raw_cov** = raw coverage of sequencing for that embryo, calculated by: ((# of reads aligned to the reference genome * 150 bp)/ 650,000,000 bp); **het_threshold** = heteroallelic match percentage cutoff for clonal embryos according to a quantile regression model; **hom_threshold** = homoallelic match percentage cutoff for clonal embryos according to a quantile regression model; **quadrant** = quadrant an embryo falls in. Q1 = Selfing or haploid parthenogenesis (fail het_threshold, pass hom_threshold). Q2 = apomixis or possible B_III_ self (pass het_threshold, pass hom_threshold). Q3 = sexual reproduction, particularly meiotic reduction, recombination, and foreign pollination (fail het_threshold, fail hom_threshold). Q4 = foreign pollination with low rates of recombination/ large haploblocks among preferred mating partners or B_III_ hybrid (pass het_threshold, fail hom_threshold).
(XLSX)

S3 Table Flow cytometry seed screen (FCSS) results for 26 *Malus* genotypes. Each row represents an independent seed analyzed. Maternal genotype = genotype a seed was derived from. Collecting season = fruiting year in which the seed was produced. Maternal ploidy = ploidy of maternal genotype. Date analyzed = date the seed was analyzed with flow cytometry. Columns with ‘Mean-x’ record the position of peaks on the x-axis and are supplemented by the coefficient of variance (CV) for the internal standard for genome size (*Carex acutiformis*; Cx), embryo (emb.), and endosperm (end.). emb/Cx = ratio of embryo to internal standard. endosperm/Cx = ratio of endosperm to internal standard. end/emb = ratio of endosperm to embryo. CV-Cx = coefficient of variance for internal standard. CV-emb = coefficient of variance for embryo. CV-end = coefficient of variance for endosperm. Embryo ploidy = ploidy of the embryo. Endosperm ploidy = ploidy of the endosperm. Ploidy of the embryo and endosperm were subsequently calculated from their peak ratios with the standard. Mating system = assignment of each seed to one of the following systems: SE = canonical sexual reproduction, AA = autonomous apomixis, PG = pseudogamy, HP = haploid parthenogenesis, GR = genome reduction, GI = genomic increase.
(XLSX)

S4 Table Summary of the subsampling results to assess consistency of threshold models. **mother** = the maternal parent an embryo is derived from; embryo = sample ID for the sequenced embryo; **total_sites** = total number of sites compared between mother and embryo in the full dataset; **total_sites_[150–2000]** = total sites compared after subsampling 150, 500, 1000, and 2000 random sites from a pruned and filtered embryo VCF. **het_sites_total** = total number of sites compared between an embryo and its maternal parent at sites where the maternal parent was heteroallelic (0/1) in the full dataset; **het_sites_total_[150–2000]** = total sites compared after subsampling 150, 500, 1000, 2000 random sites from the embryo VCF that were called heterozygous in the maternal dataset. **hom_sites_total** = total number of sites compared between an embryo and its maternal parent at sites where the maternal parent was homoallelic (0/0 or 1/1) in the full dataset; **hom_sites_total_[150–2000]** = total sites compared after subsampling 150, 500, 1000, 2000 random sites from the embryo VCF that were called homozygous in the maternal dataset. **raw_cov** = raw sequencing coverage of the embryo; **raw_cov_[150–2000]** = raw sequencing coverage of the subsampled embryo, according to the median coverage of all sequenced embryos with +/- 100 sites compared. For example, the embryos subsampled for 150 sites were assigned a coverage based on the median value of all sequenced embryos with 50–250 sites compared. The coverage is necessary to calculate the heteroallelic threshold; **original_quadrant** = reproductive quadrant the embryo was determined to fall in based on its full dataset. **quadrant_[150–2000]** = reproductive quadrant the embryo was determined to fall in based on the site level that was subsampled and compared. **num_mismatches** = number of times a quadrant was different from the original assignation across all site levels.
(XLSX)
