# Supplementary material for: Development of a low-coverage whole genome sequencing screen for apomixis using a diverse set of Malus germplasm
Source: PLoS Genet. 2026 Sep 8;22(9):e1012289. doi: 10.1371/journal.pgen.1012289 (PMC13577478; doi:10.1371/journal.pgen.1012289)
Supplement: Supplementary file 1 [file pgen.1012289.s010.docx]

Response to Reviewers

Authors responses are in green.

**Reviewer #1:** In the paper “A high-throughput sequencing screen to identify apomixis and other modes of reproduction in plants” by Goeckeritz et al., the authors use low coverage genome sequencing and flow cytometric seed screening (FCSS) from a subset of sequenced Malus (apple) species to identify variations in modes of reproduction/apomixis. Apple is a species where FCSS can be used to identify apomixis in maternal plants. The authors describe that sequencing of the maternal genome at a higher coverage along with low coverage sequencing of offspring, with careful sequencing controls, allows the identification of SNP data which can be parsed into 4 quadrants to give both apomict and additional reproductive phenotyping (ie: outcrossing vs. selfing) of the maternal line. The addition of flow cytometric seed screening can give additional reproductive information within the sequencing quadrants. The generation of numerous SNP genetic and FCSS data shows the plasticity/diversity of reproductive outcomes in Malus. While this paper is informative for apomixis in Malus, it also gives a potential pathway of analysis that could be useful for researchers studying variation for sexual and apomictic reproduction in seedlings. The authors do note that this approach for plants with larger genomes and more repetitive DNA may not continue to be price competitive.

Overall, the paper is well organized and written although some places seem overly explained (ie: the legends of Figure 3 and 4) or repetitive between the material and methods section and elsewhere in the paper.

We agree this may have made reading the manuscript more difficult. We made careful efforts to shorten and reduce figure captions (especially since the revised version’s Figure 2 and Figure 3 display similar information for different genotypes) and eliminate redundancy between the Results and Methods. However, please let us know if you feel there is still too much overlap in sections and captions.

In discussion, line 378-79 sounds like an advertisement for a kit.

This is a fair criticism, and we rephrased this to make the discussion more kit agnostic and still make our point about normalization and sample dropout.

Line 601-608 in materials is an important point and should be moved to the discussion.

Thank you for this feedback. In an earlier version of the manuscript, the inflated error rates in the tetraploid *M. platycarpa* accessions were originally discussed in both the Results and Discussion, but since the trend was only seen in these four genotypes and we feared this would distract from the rest of the findings, we abbreviated the focus on these genotypes. However, we very much agree that it’s an important point, especially in the context of choosing appropriate boundaries for quadrant determination in a diverse set of genotypes and species. Therefore, we made it a major point in the manuscript again, as researchers looking to use this method should take precautions like these to account for abnormal samples. Our initial observations of tetraploid *M. platycarpa* 589415 were also what inspired the use of spike-in controls on the embryo plates, which were crucial for modeling technical error rates.

The specific text mentioned (lines 601-608) was moved to the discussion as suggested.

Small changes:
Line 64 – need ) after parthenogenesis -- corrected
Line 79 – need ) after Meiosis -- corrected
Line 111 – remove ( before [32] -- corrected
Line 189 – year for Phipps et al. -- added
Fig 2A: move the sexual and Apomict description of the Malus phenotypes next to the name of the species in parentheses. As the figure is – it looks like those words are heads/information for the 2 quadrants on the right. – done.
Line 423-424 – PI should be removed to stay consistent with the rest of the paper. -- corrected
Line 489 degree symbol should be superscript -- corrected
Table S3 column Q – polen should be pollen and parthenogenesis – ‘h’ is missing – corrected, thank you for catching these!
- Line 244: if there is a question on type then both should be in the same column with an or between -- corrected

Thank you again for your helpful comments and review.

**Reviewer #2:** This manuscript presents a low-pass whole-genome sequencing framework to identify apomixis and other reproductive modes in Malus by directly comparing maternal and embryo genotypes. The study is potentially valuable for both reproductive biology and breeding applications. However, the novelty of this manuscript is insufficient for publication PLOS Genetics. Several aspects of the analytical framework, interpretation of the quadrants, and the handling of edge cases would benefit from further clarification or strengthening before publication.

We sincerely appreciate your feedback aimed at improving the manuscript.

1. The title is not sufficiently specific and is somewhat ambiguous.
(1) The use of “high-throughput sequencing” does not reflect the key technical feature of this study, namely low-coverage WGS.

(2) “other modes of reproduction” is too vague and should be described more specifically.
(3) “in plants” is overly broad, as the method was only tested in Malus in this study and was not validated in other species.

These are fair points - Reviewer #4 also brought (3) to our attention. While we agree the title should be more specific, we worry specifying all modes of reproduction may make the title over-bearing, and we would like to leave it somewhat open-ended as we believe the method is applicable to other genera beyond *Malus.* We found a middle ground and changed it to:

*Development of a low-coverage whole genome sequencing screen for apomixis using a diverse set of Malus germplasm*

2. As a methodological paper, a workflow diagram is suggested to summarize the study as a whole. In contrast, Fig. 3 is somewhat confusing, and its intended message is not very clear. I suggest redrawing it in the style of a graphical summary and placing it at the end of the result section or at the beginning of the discussion section.

We created a simple workflow figure and introduced it in the Discussion as **Supplementary Figure 4**. However, Figure 3 (now Figure 4 in the revised manuscript) served a different purpose - to compare the FCSS and low-sequencing methods by illustrating the types of conclusions each one provides and how they overlap. That being said, we agree the original version was somewhat overwhelming. As a compromise, we broke the figure into an A, B, and C to help explain the points it illustrates in a more piecemeal fashion and simplified the figure caption. We restructured the manuscript so that this figure fit more appropriately in the Discussion. It is now referenced in the first and fifth paragraphs of the Discussion.

3. The criteria for setting the “92.5% heteroallelic and 95% homoallelic” thresholds need further clarification. Since the 95% confidence interval was calculated, why were the boundaries lowered afterwards? These values may themselves be affected by the reference genome and the species being analyzed. Because the boundaries are key parameters for distinguishing different reproductive modes, a more practical and reproducible procedure for defining them should be provided.

These are excellent points. Our initial thresholds were admittedly somewhat arbitrary as we first explored the data. In response to this feedback, we considered regression methods using our biological and technical controls to statistically determine the clonal thresholds. Because of deviations in normality and heteroskedasticity in the dataset, we settled on using quantile regression to model the genetic similarity thresholds that 95% of true clonal embryos were expected to surpass. We hope that you will find that these methods are more reproducible. Please see sections ‘Establishing clonal thresholds with quantile regression’ in the Results section and ‘Statistical determination of clonal boundary cutoffs with biological and technical controls (spike-ins)’ in the Materials and Methods for further details.

4. It is not clear how sample ploidy was determined, whether through WGS data or flow cytometry.

Maternal genotypes screened in our study were previously assessed with nuclear DNA flow cytometry, as stated in the Methods:

*“The ploidy level of each maternal genotype was determined previously by NPGS with flow cytometry and is listed with other characteristics of the accession in the GRIN-Global database* [61].*”*

USDA-NPGS (United States Department of Agriculture - National Plant Germplasm System) researchers outsourced the collection of this data for 1651 *Malus* in their collection in 2008 according to curator records, and the service was performed by the Plant Cytometry Service in the Netherlands: <https://npgsweb.ars-grin.gov/gringlobal/method?id=494854>. However, a formal publication for these methods was not made available, thus a general reference for the germplasm database was used (Byrne *et al*. 2018).

5. Please check Fig. S1, as the legend contains “NA”.

Thank you so much for catching this. This was due to a coding error in R that prevented the proper labeling of *M. platycarpa* 589415. It was corrected - however, since this figure was somewhat redundant with **Figure 1B**, we decided to exclude it from the revised manuscript.

6. Line 312: I could not clearly identify the 21 facultative apomicts or the 9 genotypes complemented with FCSS. Please highlight them more clearly in Fig. 4.

Thank you for highlighting an important fact: the original version of Figure 4 (now **Figure 3** in the revised manuscript) lacked a cohesive structure which made it hard to find specific details. Actually, developing the statistical framework for determining clonal boundary cutoffs also prompted a redesign of this figure. We hope this will make it easier to identify the facultative apomicts (these genotypes’ subplots have at least one open green circle near the top right corner, and green coloration in their pies) and which genotypes were complemented with FCSS at a glance (asterisk in the bottom right corner of each subplot).

**Reviewer #3**: The manuscript by Goeckeritz and colleagues presents a strategy to analyze reproduction modes in plants with a particular interest for identifying species and accession that produce apomictic seeds. The strategy relies on low-depth whole-genome sequencing that nevertheless still provides enough informative variant sites to infer reproduction modes after comparing maternal and offspring genotypes. The method appears sound, with carefully controlled thresholds for identifying high confidence SNP sites. In addition to presenting the pipeline to execute this method, the authors used it to screen a large number of Malus genotypes as a proof of concept, and they identified several genotypes showing facultative apomixis. Finally, they compared the results of their screening with a previously established method for inferring reproduction modes (FCSS). Together, the presented strategy appears to be a promising tool that will be useful for the plant sexual reproduction community.

Thank you very much for your time in reviewing the manuscript, and we are very pleased to hear that you feel this could be a useful strategy for plant reproductive biologists.

I have only a few questions:

1) The authors show average coverage for each of their samples in Fig 1. What is the average coverage in these samples at informative variant sites? I assume the coverage at such a site has to be higher for it to be confidently called?

Yes, absolutely! For the low-coverage embryo data, sites throughout the genome will be covered on average at the levels shown in **Figure 1A** and **1B** for each embryo. Of course, some sites have no coverage and others have much higher coverage – sites passing our thresholds for variant calling fall into the latter category. This excerpt is from the methods of our manuscript to help answer your question:

*Low (LDP) and high depth (HDP) filters, or the minimum and maximum number of reads supporting a site, were set according to the ploidy of the maternal parent and the embryo’s approximate coverage. For embryos of diploid, triploid, and tetraploid parents, a minimum of 10, 11, and 12 reads, respectively, were required to support a genotype call. For embryos with average coverages of 1X, 2X, 3X - 6X, and > 6X, the maximum number of reads allowed for any given call was 25, 30, 30, and 40, respectively.*

Therefore, while we did not calculate average coverage at all compared sites, we do know each site’s coverage lies somewhere between 10 and 40 reads for all embryo samples.

2) For accessions with deep whole genome sequencing data available, what is the total number of variant sites compared to the number of variant sites that pass the threshold in low coverage data?

There are a few potential ways to interpret this question. If you mean how many sites passed filtering in the maternal genotypes (these were sequenced to ~15X or deeper) and how many of those were found in the low coverage embryo files that also passed filtering, then this information can be found in **Table S2** for every embryo:maternal_parent comparison (granted the embryo passed the initial filter of having at least 50 het and 50 hom sites to compare). The column total_sites in this table gives the number of all sites compared between the embryo and maternal parent for that row, and there is also a het_sites_total and hom_sites_total that breaks down which of those sites in the maternal parent were heterozygous and homozygous, respectively. If this does not answer your question, please clarify and we’ll do our best to answer.

3) For the vast majority of analyzed embryos, thousands of variant sites were available. How robust are the % matching Het/Hom site numbers with regards to the number of informative sites? Would a random selection of 100 sites lead to the same conclusions with regards to the position of the embryo in the quadrants? If not, what is the minimal number of sites needed?

This is a very thoughtful and much appreciated question. To answer it, we subsampled some of the pruned, filtered embryo vcf files at several site levels, then checked the consistency of our quadrant determinations at that site level compared to the full dataset. For an embryo to be eligible for subsampling, its full dataset had to have at least 5000 sites compared with its maternal parent. We selected 3 random embryos from 20 random mothers (9 diploids, 7 triploids, 4 tetraploids), then sampled 150, 500, 1000, and 2000 random sites for merging with the maternal vcf and comparing with bcftools gtcheck. (**Note**: we started the subsampling at 150 because there are even some sites in the embryos that could be absent in the maternal files, so to get close to your 100 site question, we sampled a little higher. The range and mean of sites compared for the 150 site level was 83 - 134, and 115, respectively). The results of the subsampling were written into a section of the Results right after the quadrant descriptions:

*To evaluate how our models performed with fewer compared sites (mainly a consequence of low sequencing coverage), we subsampled sites from 3 embryos representing 20 maternal genotypes that each had ≥ 5000 compared sites in their complete datasets (****Table S4****). Pruned embryo vcfs were subsampled for 150, 500, 1000, and 2000 random sites and assigned a coverage value based on the median coverage for all embryos with ± 100 sites (0.3X, 0.5X, 0.6X, and 0.9X coverage, respectively). For the 150 and 500 site comparisons, 50/60 of the embryos were assigned the same reproductive quadrant as their full datasets. For the 1000 and 2000 site comparisons, 53/60 and 56/60 were assigned identical quadrants, respectively. Most mismatches within each subsampling level represented shifts in sexual classifications (Q1, Q3, and Q4) rather than shifts in asexuality to sexuality and vice versa (Q2 → other or other → Q2). For the latter scenarios specifically, the 150, 500, 1000, and 2000 had four, four, two, and zero embryos involving Q2 misclassifications. Taken together, these results demonstrate accuracies of 83.3 – 93.3 % across all sequencing quadrants even when comparing very few sites, and higher accuracies (93.3 – 100 %) when simply distinguishing clonally- versus sexually-produced embryos.*

We also added **Table S4** to the manuscript to summarize the outcomes of the subsampling. We hope this answers your question in full.

4) What guidelines would the authors offer when adapting this pipeline for other species that potentially have different levels of genetic variation? What is the minimum number of variant sites required (similar to point 3)? Knowing the standing genetic variation, what number of informative sites in low-depth sequencing can be expected if one knows the total number of potential sites (similar to point 2)?

These are great thought-provoking questions. We added some text to the discussion for researchers thinking to adapt this method to another genus / group - which will depend on the species’ biology. Here is the Discussion section excerpt we had in mind for your question:

*Ploidy, genome size, amenability to FCSS, reference genome availability, breeding tendencies, allele frequencies, and heterozygosity levels should all be considered when designing low-pass screening-by-sequencing experiments. For instance, heterozygous outcrossing species (including Malus) would be expected to require less sequencing coverage for robust conclusions compared to highly inbred populations, as more informative sites would be captured by chance. A small pilot experiment including several genotypes representing the range of diversity for a particular system may be necessary before expanding the scale of screening. For larger genomes, imputation may assist with data sparseness* [*[65]*](https://paperpile.com/c/APke7f/TpyO)*, or a custom probe set coupled with enrichment sequencing would ensure adequate coverage of polymorphic locations that are evenly distributed across the genome – however, this approach would require the design of species- or genera-specific markers* [*[66]*](https://paperpile.com/c/APke7f/j5Rx)*.*

As for any variant calling pipeline, we also want to point out that the number of variants found may fluctuate depending on the reference genome a researcher decides to use - although the overall conclusions (asexual vs sexual reproduction, for instance) should be stable. While it is difficult to give an exact amount of needed sites given the many factors influencing this, having a general understanding of population diversity from the literature and breeding tendencies (e.g., predominant selfing or outcrossing) would be the best place to start when designing an initial pilot study.

We hope we have answered all your questions and genuinely appreciate you stretching our thinking and helping us improve the manuscript.

**Reviewer #4:** Generally the authors developed a very efficient strategy to identify apomixis from embryos. The method is very important to identify types of reproduction. I would offer several suggestions to improve the manuscript.
1. the title need to change. It seems the authors only test this method in apple. So the title should precisely reflect the conclusion in this study.

We have now changed the title to:

*Development of a low-coverage whole genome sequencing screen for apomixis using a diverse set of Malus germplasm*

2. The identified plants with apomixis need to be confirmed by PCR or allele-specific markers.

We chose not to do this type of confirmation, given that we generated thousands of SNP markers through whole genome shotgun resequencing of these embryos, which is much higher resolution than a small panel of PCR markers.

3. Abstract Line 40, four of these were detected using internal sequencing controls. It is not clear.

We can see how this would be a confusing thing to place in the abstract as a standalone term. This was originally referring to our spike-ins’ abilities to identify the higher error rates in the four tetraploid *M. platycarpa* samples. However, after statistical determination of the quadrants we concluded FCSS may be the best method to determine the mode of reproduction in some genotypes where error rate was difficult to model in low-coverage datasets. We edited the abstract text to reflect these changes. The sentence referenced above was specifically changed to:

*Several of these were detected with the flow cytometry seed screen, which helped resolve certain types of reproduction and sources of noise in low-coverage datasets.*

We want to thank you again for your helpful comments on the manuscript.
