# Supplementary material for: Development of a low-coverage whole genome sequencing screen for apomixis using a diverse set of Malus germplasm
Source: PLoS Genet. 2026 Sep 8;22(9):e1012289. doi: 10.1371/journal.pgen.1012289 (PMC13577478; doi:10.1371/journal.pgen.1012289)
Supplement: Peer review history

# Peer review: Decision Letter 0

**DOI:** 10.1371/journal.pgen.1012289.r001

**Authors:** Jae Young Choi, Quan Wang

9 May 2026

PGENETICS-D-26-00294

A high-throughput sequencing screen to identify apomixis and other modes of reproduction in plants

PLOS Genetics

Dear Dr. Harkess,

Thank you for submitting your manuscript to PLOS Genetics. After careful consideration, we feel that it has merit but does not fully meet PLOS Genetics's publication criteria as it currently stands. Therefore, we invite you to submit a revised version of the manuscript that addresses the points raised during the review process.

Please submit your revised manuscript by . If you will need more time than this to complete your revisions, please reply to this message or contact the journal office at plosgenetics@plos.org. When you're ready to submit your revision, log on to https://www.editorialmanager.com/pgenetics/ and select the 'Submissions Needing Revision' folder to locate your manuscript file.

Please include the following items when submitting your revised manuscript:

* A letter that responds to each point raised by the editor and reviewer(s). You should upload this letter as a separate file labeled 'Response to Reviewers'. This file does not need to include responses to any formatting updates and technical items listed in the 'Journal Requirements' section below.

* A marked-up copy of your manuscript that highlights changes made to the original version. You should upload this as a separate file labeled 'Revised Manuscript with Track Changes'.

* An unmarked version of your revised paper without tracked changes. You should upload this as a separate file labeled 'Manuscript'.

If you would like to make changes to your financial disclosure, competing interests statement, or data availability statement, please make these updates within the submission form at the time of resubmission. Guidelines for resubmitting your figure files are available below the reviewer comments at the end of this letter.

As the corresponding author, your ORCID iD is verified in the submission system and will appear in the published article. PLOS supports the use of ORCID, and we encourage all coauthors to register for an ORCID iD and use it as well. Please encourage your coauthors to verify their ORCID iD within the submission system before final acceptance, as unverified ORCID iDs will not appear in the published article. Only the individual author can complete the verification step; PLOS staff cannot verify ORCID iDs on behalf of authors.

We look forward to receiving your revised manuscript.

Kind regards,

Jae Young Choi

Academic Editor

PLOS Genetics

Quan Wang

Section Editor

PLOS Genetics

Aimée Dudley

Editor-in-Chief

PLOS Genetics

Anne Goriely

Editor-in-Chief

PLOS Genetics

**Additional Editor Comments:**

The manuscript has been reviewed by four reviewers. The reviewers have found the topic of the study and results interesting, and three of the reviewers were generally positive with the paper. However one reviewer (reviewer 2) does have some concerns that addressing it would greatly improve the current paper. Based on the reviews, we will not be able to accept this version of the manuscript, but we would be willing to review a much-revised version that fully addresses each of the points raised by the reviewers.

**Journal Requirements:**

1) We do not publish any copyright or trademark symbols that usually accompany proprietary names, eg ©,  ®, or TM  (e.g. next to drug or reagent names). Therefore please remove all instances of trademark/copyright symbols throughout the text, including:

- ® on page: 22 and 23.

- TM on page: 17..

2) Please upload all main figures as separate Figure files in .tif or .eps format. For more information about how to convert and format your figure files please see our guidelines:

https://journals.plos.org/plosgenetics/s/figures

3) Please amend your detailed Financial Disclosure statement. This is published with the article. It must therefore be completed in full sentences and contain the exact wording you wish to be published.

- State the initials, alongside each funding source, of each author to receive each grant. For example: "This work was supported by the National Institutes of Health (####### to AM; ###### to CJ) and the National Science Foundation (###### to AM)."

- State what role the funders took in the study. If the funders had no role in your study, please state: "The funders had no role in study design, data collection and analysis, decision to publish, or preparation of the manuscript.".

If you did not receive any funding for this study, please simply state: u201cThe authors received no specific funding for this work.u201d

**Reviewers' comments:**

Reviewer's Responses to Questions

**Comments to the Authors:**

**Please note here if the review is uploaded as an attachment.**

Reviewer #1: In the paper “A high-throughput sequencing screen to identify apomixis and other modes of reproduction in plants” by Goeckeritz et al., the authors use low coverage genome sequencing and flow cytometric seed screening (FCSS) from a subset of sequenced Malus (apple) species to identify variations in modes of reproduction/apomixis. Apple is a species where FCSS can be used to identify apomixis in maternal plants. The authors describe that sequencing of the maternal genome at a higher coverage along with low coverage sequencing of offsping, with careful sequencing controls, allows the identification of SNP data which can be parsed into 4 quadrants to give both apomict and additional reproductive phenotyping (ie: outcrossing vs. selfing) of the maternal line. The addition of flow cytometric seed screening can give additional reproductive information within the sequencing quadrants. The generation of numerous SNP genetic and FCSS data shows the plasticity/diversity of reproductive outcomes in Malus. While this paper is informative for apomixis in Malus, it also gives a potential pathway of analysis that could be useful for researchers studying variation for sexual and apomictic reproduction in seedlings. The authors do note that this approach for plants with larger genomes and more repetitive DNA may not continue to be price competitive. Overall, the paper is well organized and written although some places seem overly explained (ie: the legends of Figure 3 and 4) or repetitive between the material and methods section and elsewhere in the paper. In discussion, line 378-79 sounds like an advertisement for a kit. Line 601-608 in materials is an important point and should be moved to the discussion.

Small changes:

Line 64 – need ) after parthenogenesis

Line 79 – need ) after Meiosis

Line 111 – remove ( before [32]

Line 189 – year for Phipps et al.

Fig 2A: move the sexual and Apomict description of the Malus phenotypes next to the name of the species in parentheses. As the figure is – it looks like those words are heads/information for the 2 quadrants on the right.

Line 423-424 – PI should be removed to stay consistent with the rest of the paper.

Line 489 degree symbol should be superscript

Table S3 column Q – polen should be pollen and parthenogenesis – ‘h’ is missing

- Line 244: if there is a question on type then both should be in the same column with an or between

Reviewer #2: This manuscript presents a low-pass whole-genome sequencing framework to identify apomixis and other reproductive modes in Malus by directly comparing maternal and embryo genotypes. The study is potentially valuable for both reproductive biology and breeding applications. However, the novelty of this manuscript is insufficient for publication PLOS Genetics. Several aspects of the analytical framework, interpretation of the quadrants, and the handling of edge cases would benefit from further clarification or strengthening before publication.

1. The title is not sufficiently specific and is somewhat ambiguous.

(1) The use of “high-throughput sequencing” does not reflect the key technical feature of this study, namely low-coverage WGS.

(2) “other modes of reproduction” is too vague and should be described more specifically.

(3) “in plants” is overly broad, as the method was only tested in Malus in this study and was not validated in other species.

2. As a methodological paper, a workflow diagram is suggested to summarize the study as a whole. In contrast, Fig. 3 is somewhat confusing, and its intended message is not very clear. I suggest redrawing it in the style of a graphical summary and placing it at the end of the result section or at the beginning of the discussion section.

3. The criteria for setting the “92.5% heteroallelic and 95% homoallelic” thresholds need further clarification. Since the 95% confidence interval was calculated, why were the boundaries lowered afterwards? These values may themselves be affected by the reference genome and the species being analyzed. Because the boundaries are key parameters for distinguishing different reproductive modes, a more practical and reproducible procedure for defining them should be provided.

4. It is not clear how sample ploidy was determined, whether through WGS data or flow cytometry.

5. Please check Fig. S1, as the legend contains “NA”.

6. Line 312: I could not clearly identify the 21 facultative apomicts or the 9 genotypes complemented with FCSS. Please highlight them more clearly in Fig. 4.

Reviewer #3: The manuscript by Goeckeritz and colleagues presents a strategy to analyze reproduction modes in plants with a particular interest for identifying species and accession that produce apomictic seeds. The strategy relies on low-depth whole-genome sequencing that nevertheless still provides enough informative variant sites to infer reproduction modes after comparing maternal and offspring genotypes. The method appears sound, with carefully controlled thresholds for identifying high confidence SNP sites. In addition to presenting the pipeline to execute this method, the authors used it to screen a large number of Malus genotypes as a proof of concept, and they identified several genotypes showing facultative apomixis. Finally, they compared the results of their screening with a previously established method for inferring reproduction modes (FCSS). Together, the presented strategy appears to be a promising tool that will be useful for the plant sexual reproduction community.

I have only a few questions:

1) The authors show average coverage for each of their samples in Fig 1. What is the average coverage in these samples at informative variant sites? I assume the coverage at such a site has to be higher for it to be confidently called?

2) For accessions with deep whole genome sequencing data available, what is the total number of variant sites compared to the number of variants sites that pass the threshold in low coverage data?

3) For the vast majority of analyzed embryos, thousands of variant sites were available. How robust are the % matching Het/Hom site numbers with regards to the number of informative sites? Would a random selection of 100 sites lead to the same conclusions with regards to the position of the embryo in the quadrants? If not, what is the minimal number of sites needed?

4) What guidelines would the authors offer when adapting this pipeline for other species that potentially have different levels of genetic variation? What is the minimum number of variant sites required (similar to point 3)? Knowing the standing genetic variation, what number of informative sites in low-depth sequencing can be expected if one knows the total number of potential sites (similar to point 2)?

Reviewer #4: Generally the authors developed a very efficient strategy to identify apomixis from embyos. The method is very important to identify typs of reproduction. I would offer serveral suggestions to improve the manuscript.

1. the title need to change. It seems the authors only test this method in apple. So the title should precisely reflect the conclusion in this study.

2. The identifed plants with apomixis need to be confirmed by PCR or allele-specific markers.

3. Abstract Line 40, four of these were detected using internal sequencing controls. It is not clear.

**********

**Have all data underlying the figures and results presented in the manuscript been provided?**

Large-scale datasets should be made available via a public repository as described in the *PLOS Genetics*  data availability policy, and numerical data that underlies graphs or summary statistics should be provided in spreadsheet form as supporting information.

Reviewer #1: Yes

Reviewer #2: None

Reviewer #3: None

Reviewer #4: None

**********

PLOS authors have the option to publish the peer review history of their article (what does this mean?). If published, this will include your full peer review and any attached files.

If you choose “no”, your identity will remain anonymous but your review may still be made public.

**Do you want your identity to be public for this peer review?**  For information about this choice, including consent withdrawal, please see our Privacy Policy.

Reviewer #1: No

Reviewer #2: No

Reviewer #3: No

Reviewer #4: No

[NOTE: If reviewer comments were submitted as an attachment file, they will be attached to this email and accessible via the submission site. Please log into your account, locate the manuscript record, and check for the action link "View Attachments". If this link does not appear, there are no attachment files.]

**Figure resubmission:**

While revising your submission, we strongly recommend that you use PLOS’s NAAS tool (https://ngplosjournals.pagemajik.ai/artanalysis) to test your figure files. NAAS can convert your figure files to the TIFF file type and meet basic requirements (such as print size, resolution), or provide you with a report on issues that do not meet our requirements and that NAAS cannot fix.

After uploading your figures to PLOS’s NAAS tool - https://ngplosjournals.pagemajik.ai/artanalysis, NAAS will process the files provided and display the results in the "Uploaded Files" section of the page as the processing is complete. If the uploaded figures meet our requirements (or NAAS is able to fix the files to meet our requirements), the figure will be marked as "fixed" above. If NAAS is unable to fix the files, a red "failed" label will appear above. When NAAS has confirmed that the figure files meet our requirements, please download the file via the download option, and include these NAAS processed figure files when submitting your revised manuscript.

**Reproducibility:**

To enhance the reproducibility of your results, we recommend that authors of applicable studies deposit laboratory protocols in protocols.io, where a protocol can be assigned its own identifier (DOI) such that it can be cited independently in the future. Additionally, PLOS ONE offers an option to publish peer-reviewed clinical study protocols. Read more information on sharing protocols at https://plos.org/protocols?utm_medium=editorial-email&utm_source=authorletters&utm_campaign=protocols

# Author comment: Author response to Decision Letter 1

**DOI:** 10.1371/journal.pgen.1012289.r002

23 Jul 2026

Attachment Submitted filename: Response to Reviewers.docx

# Peer review: Decision Letter 1

**DOI:** 10.1371/journal.pgen.1012289.r003

**Authors:** Jae Young Choi, Quan Wang

17 Aug 2026

Dear Dr Harkess,

We are pleased to inform you that your manuscript entitled ": Development of a low-coverage whole genome sequencing screen for apomixis using a diverse set of Malus germplasm" has been editorially accepted for publication in PLOS Genetics. Congratulations!

Before your submission can be formally accepted and sent to production you will need to complete our formatting changes, which you will receive in a follow up email. Please be aware that it may take several days for you to receive this email; during this time no action is required by you. Please note: the accept date on your published article will reflect the date of this provisional acceptance, but your manuscript will not be scheduled for publication until the required changes have been made.

Once your paper is formally accepted, an uncorrected proof of your manuscript will be published online ahead of the final version, unless you’ve already opted out via the online submission form. If, for any reason, you do not want an earlier version of your manuscript published online or are unsure if you have already indicated as such, please let the journal staff know immediately at plosgenetics@plos.org.

In the meantime, please log into Editorial Manager at https://www.editorialmanager.com/pgenetics/, click the "Update My Information" link at the top of the page, and update your user information to ensure an efficient production and billing process. Note that PLOS requires an ORCID iD for all corresponding authors. Therefore, please ensure that you have an ORCID iD and that it is validated in Editorial Manager. To do this, go to ‘Update my Information’ (in the upper left-hand corner of the main menu), and click on the Fetch/Validate link next to the ORCID field.  This will take you to the ORCID site and allow you to create a new iD or authenticate a pre-existing iD in Editorial Manager.

If you have a press-related query, or would like to know about making your underlying data available (as you will be aware, this is required for publication), please see the end of this email. If your institution or institutions have a press office, please notify them about your upcoming article at this point, to enable them to help maximise its impact. Inform journal staff as soon as possible if you are preparing a press release for your article and need a publication date.

Thank you again for supporting open-access publishing; we are looking forward to publishing your work in PLOS Genetics!

Yours sincerely,

Jae Young Choi

Academic Editor

PLOS Genetics

Quan Wang

Section Editor

PLOS Genetics

Aimée Dudley

Editor-in-Chief

PLOS Genetics

Anne Goriely

Editor-in-Chief

PLOS Genetics

www.plosgenetics.org

BlueSky: @plos.bsky.social

----------------------------------------------------

Comments from the reviewers (if applicable):

Reviewer's Responses to Questions

**Comments to the Authors:**

**Please note here if the review is uploaded as an attachment.**

Reviewer #1: The revised manuscript "Development of a low-coverage whole genome sequencing screen for apomixis using

a diverse set of Malus germplasm" uses low coverage sequencing, optimized modeling and flow cytometry of seed to identify Malus accessions which reproduce by apomixis. Their data include previously defined sexual and apomictic accessions as controls along with screening accessions with unknown reproduction or that are potentially classified as apomicts in the literature. As a revised manuscript, the authors took care to reply to the first set of reviewers and have made significant changes to the manuscript based on those suggestions to strengthen the manuscript.

Reviewer #3: In their revised manuscript, Goeckeritz and colleagues improved the clarity of the presentation and the description of the methodology underlying their proposed approach to characterize sexual/apomictic modes of reproduction through a combination of low-coverage whole genome sequencing and flow cytometry. They addressed all my questions in a satisfactory manner. I have no further comments.

Reviewer #4: The authors addressed my concern.

**********

**Have all data underlying the figures and results presented in the manuscript been provided?**

Large-scale datasets should be made available via a public repository as described in the *PLOS Genetics*  data availability policy, and numerical data that underlies graphs or summary statistics should be provided in spreadsheet form as supporting information.

Reviewer #1: Yes

Reviewer #3: None

Reviewer #4: None

**********

PLOS authors have the option to publish the peer review history of their article (what does this mean?). If published, this will include your full peer review and any attached files.

If you choose “no”, your identity will remain anonymous but your review may still be made public.

**Do you want your identity to be public for this peer review?**  For information about this choice, including consent withdrawal, please see our Privacy Policy.

Reviewer #1: No

Reviewer #3: No

Reviewer #4: No

----------------------------------------------------

**Data Deposition**

If you have submitted a Research Article or Front Matter that has associated data that are not suitable for deposition in a subject-specific public repository (such as GenBank or ArrayExpress), one way to make that data available is to deposit it in the Dryad Digital Repository. As you may recall, we ask all authors to agree to make data available; this is one way to achieve that. A full list of recommended repositories can be found on our website.

The following link will take you to the Dryad record for your article, so you won't have to re‐enter its bibliographic information, and can upload your files directly:

http://datadryad.org/submit?journalID=pgenetics&manu=PGENETICS-D-26-00294R1

More information about depositing data in Dryad is available at http://www.datadryad.org/depositing. If you experience any difficulties in submitting your data, please contact help@datadryad.org for support.

Additionally, please be aware that our data availability policy requires that all numerical data underlying display items are included with the submission, and you will need to provide this before we can formally accept your manuscript, if not already present.

----------------------------------------------------

**Press Queries**

If you or your institution will be preparing press materials for this manuscript, or if you need to know your paper's publication date for media purposes, please inform the journal staff as soon as possible so that your submission can be scheduled accordingly. Your manuscript will remain under a strict press embargo until the publication date and time. This means an early version of your manuscript will not be published ahead of your final version. PLOS Genetics may also choose to issue a press release for your article. If there's anything the journal should know or you'd like more information, please get in touch via plosgenetics@plos.org.

# Editor report: Acceptance letter

**DOI:** 10.1371/journal.pgen.1012289.r004

**Authors:** Jae Young Choi, Quan Wang

PGENETICS-D-26-00294R1

: Development of a low-coverage whole genome sequencing screen for apomixis using a diverse set of Malus germplasm

Dear Dr Harkess,

We are pleased to inform you that your manuscript entitled ": Development of a low-coverage whole genome sequencing screen for apomixis using a diverse set of Malus germplasm" has been formally accepted for publication in PLOS Genetics! Your manuscript is now with our production department and you will be notified of the publication date in due course.

The corresponding author will soon be receiving a typeset proof for review, to ensure errors have not been introduced during production. Please review the PDF proof of your manuscript carefully, as this is the last chance to correct any errors. Please note that major changes, or those which affect the scientific understanding of the work, will likely cause delays to the publication date of your manuscript.

Soon after your final files are uploaded, unless you have opted out or your manuscript is a front-matter piece, the early version of your manuscript will be published online. The date of the early version will be your article's publication date. The final article will be published to the same URL, and all versions of the paper will be accessible to readers.

For Research Articles, you will receive an invoice from PLOS for your publication fee after your manuscript has reached the completed accept phase. If you receive an email requesting payment before acceptance or for any other service, this may be a phishing scheme. Learn how to identify phishing emails and protect your accounts at https://explore.plos.org/phishing.

Thank you again for supporting PLOS Genetics and open-access publishing. We are looking forward to publishing your work!

With kind regards,

Janani Seenivasan

PLOS Genetics

On behalf of:

The PLOS Genetics Team

Carlyle House, Carlyle Road, Cambridge CB4 3DN | United Kingdom

plosgenetics@plos.org | +44 (0) 1223-442823

plosgenetics.org | Twitter: @PLOSGenetics
